# Quantitative investigation and intelligent forecasting of thermal conductivity in lime-modified red clay

**DOI:** 10.1371/journal.pone.0311882

**Published:** 2024-10-10

**Authors:** Hongqi Wang, Dongwei Li, Zecheng Wang, Zhiwen Jia, Zhenhua Wang

**Affiliations:** 1 School of Civil and Architectural Engineering, East China University of Technology, NanChang, China; 2 School of Civil Engineering, Dalian University, Dalian, China; Beijing University of Technology, CHINA

## Abstract

This paper delves into the engineering applications of lime-stabilized red clay, a highly water-sensitive material, particularly in the context of the climatic conditions prevalent in the Dalian region. We systematically investigate the impact of water content, dry density, and freeze-thaw cycles (with a freezing temperature set at -10°C) on the thermal conductivity of stabilized soil, a crucial parameter for analyzing soil temperature fields that is influenced by numerous factors. By developing and validating both empirical and machine learning prediction models, we unravel the evolution of thermal conductivity in response to these factors: within the range of influencing variables, thermal conductivity exhibits an exponential or linear increase with rising water content and dry density, while it decreases exponentially with increasing freeze-thaw cycles. Furthermore, we quantitatively analyze the specific influence of water content and other factors on the thermal conductivity of stabilized soil and construct a comprehensive prediction model encompassing BP neural network, gradient boosting decision tree, and linear regression models. Comparative analysis highlights the significant enhancement in prediction accuracy achieved by the proposed ensemble model over single machine learning models, with root mean square error (RMSE) values below 0.05 and mean absolute percentage error (MAPE) values remaining under 2.5% in both frozen and unfrozen states. Additionally, a secondary validation using experimental data from other researchers confirms the model’s good agreement with previous results, demonstrating its robust generalization ability. Our findings provide valuable insights for engineering studies in the Dalian region and red clay areas subjected to extreme climatic conditions.

## 1. Introduction

Red clay, prevalent in southwestern and northeastern China, is routinely enhanced with cement, lime, and other additives to enhance its mechanical properties for engineering applications. While extensive research has delved into the stress field of lime-modified red clay, investigations into its temperature field remain limited. Thermal conductivity, a pivotal parameter in the temperature field, characterizes the heat transfer capability of materials. Thus, exploring the thermal conductivity of lime-modified red clay is crucial for elucidating macro-scale issues encountered in engineering applications from a temperature field perspective, facilitating further study and analysis of temperature field characteristics.

Against this backdrop, Qilin G et al. [1] examined the evolution of thermal conductivity in frozen clay soil with respect to water content and dry density under extreme climatic conditions in southern regions, revealing its variation patterns. Bo C [2] focused on Guangxi red clay, exploring the thermal conductivity during the drying process, elucidating its evolution based on three-phase transformation. Zhenhui S [3] analyzed the correlation between thermal conductivity and matrix suction, highlighting the intricate link between saturation and the three-phase relationship. Zhenhui S conducted a comprehensive analysis of soil heat transfer theory, discussing the impact of varying saturation levels on the thermal conductivity of red clay.

Shuanghui T and Yudong W [4, 5] investigated the evolution of thermal conductivity in red clay related to bound water content, emphasizing the influence of water content and bound water content on soil thermal conductivity, offering a novel perspective. Yunshan X et al. [6] studied the relationship between water content and thermal conductivity in expansive soil and red clay, using two types of high liquid limit soil as specimens. They modified the previous formula model based on boundary water content, enhancing predictive capabilities. Zhaotian Zeng et al. [7] analyzed the correlation between thermal conductivity and water content in four types of Guangxi red clay, developing a theoretical prediction model integrating composite medium theory and the parallel method, providing a novel approach. Rui T and Hongqi W et al. [8, 9] studied the evolution of soil thermal conductivity in negative temperature states, based on the influence of temperature and other factors on permafrost thermal conductivity, while Wang achieved accurate predictions of soil thermal conductivity in low-temperature states through various machine learning models. Xiangtian X and Xiuling Rui et al. [10, 11] investigated the change in soil thermal conductivity during the transition from positive to negative temperatures, constructing a machine learning model for precise predictions during freezing.

Collectively, numerous scholars have investigated the thermal conductivity of red clay, considering various factors including water content, and formulated corresponding prediction models based on formulaic theory, advancing the field. However, given the unique properties of red clay and its frequent utilization in engineering post-improvement, research on the thermal conductivity of improved soil remains scarce, indicating ample potential for further exploration. Additionally, the formulaic model of thermal conductivity under multifactor coupling requires further validation and refinement. The utilization of machine learning models in thermal conductivity research is limited but holds significant value in advancing research and prediction capabilities.

Hence, based on the ambient temperature in the Dalian region, this paper sets the minimum temperature of -10°C as the freezing temperature and room temperature as the thawing temperature to study the thermal conductivity of lime-modified red clay under the influence of freeze-thaw cycles, water content, dry density, and other factors. An empirical model is formulated to quantitatively analyze the thermal conductivity of the improved soil, considering the combined effects of various factors. Furthermore, multiple intelligent prediction models are developed to forecast and evaluate the conductivity, aiming to provide valuable insights for engineering challenges in red clay regions.

## 2. Test materials and methods

### 2.1 Test materials and scheme

The fundamental physical properties of the red clay utilized in the experimental study are presented in Table 1.

**Table 1:** Basic physical parameters of red clay.

Soil samples	Optimal water content /(%)	Maximum Dry density /(g·cm^−3^)	Plastic limit /(%)	Liquid limit /(%)	Proportion
Red clay.	21.33	1.66	17.26	45.99	2.74

A comprehensive full factorial test was conducted, encompassing three critical factors: water content, dry density, and freeze-thaw cycles. The experimental design, outlined in Table 2, entailed five distinct levels for each factor, ensuring a rigorous assessment of their individual and combined effects.

**Table 2:** Test plan.

Soil samples	Water content/(%)	Dry density /(g·cm^−3^)	Freeze-thaw cycle. /(Times)	Freeze-thaw temperature. /(°C)
Lime improved red clay ^①^	19 21 23 25 27	1.2 1.3 1.4 1.5 1.6	1 3 5 7 9	-10°C 20°C

Note:

^①^In this study, the lime content in the research process was determined based on the optimal lime content for improving the stress field of red clay, as previously investigated by researchers [12–14].

^②^ In which the SEM electron microscope scanning test was carried out using a specimen with 21% water content and 1.6 g·cm^−3^ dry density to observe the pore changes of the specimen during the freeze-thaw cycle.

### 2.2 Test method and process

**Fig 1:**
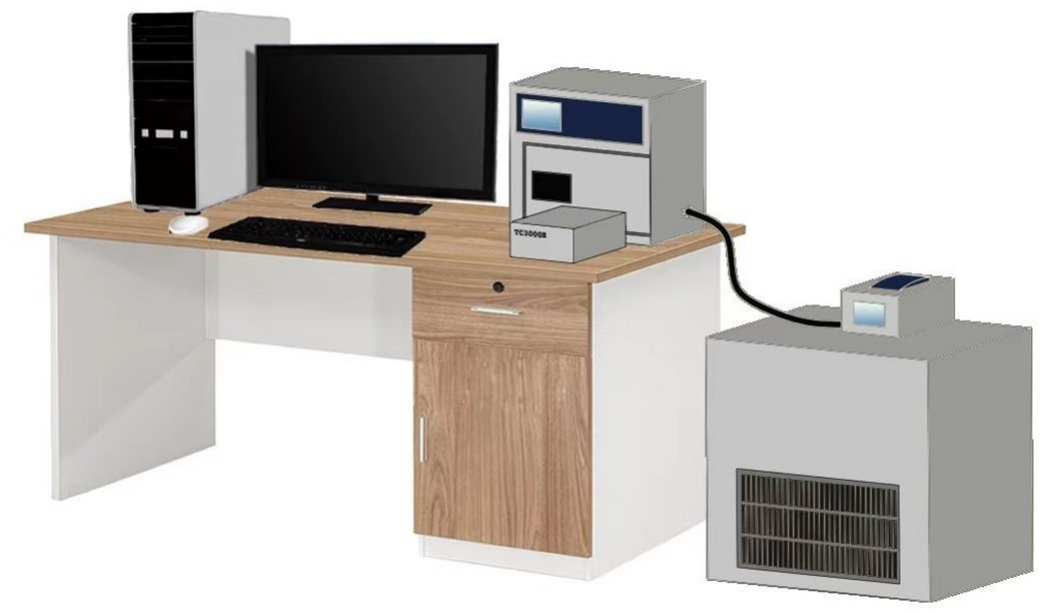
Test instrument.

A comprehensive factorial analysis of the thermal conductivity of lime-modified red clay, considering the effects of freeze-thaw cycles, water content, and dry density, was conducted utilizing a transient hot wire thermal conductivity meter. Fig 1 depicts the test instrument used in this study.

As shown in Fig 1, the test instrument mainly consists of a computer, Xiaxi TC3000E transient hot-wire method thermal conductivity meter, as well as a set of low-temperature environment box and a cold bath box independently. Among them, the cold bath mainly provides refrigerant for the low-temperature environment box to keep the inside of the low-temperature environment box at the set ambient temperature. The cryogenic testing process is carried out in the cryogenic chamber.

The preparation and testing procedures for the soil samples are outlined as follows:

1. Mixing of Materials: Quicklime is initially added to dried red clay in a preset ratio of 6%, and the mixture is thoroughly stirred to ensure homogeneity.
2. Water Addition and Sealing: A predetermined amount of water is uniformly sprayed onto the mixed soil to achieve the desired water content. The soil is then sealed for 24 hours to ensure uniform water distribution within the sample.
3. Sample Preparation: Based on the preset dry density, the wet density is calculated and the soil is weighed accordingly. A ring knife mold of dimensions φ61.8mm×20mm is utilized to prepare a standard soil sample, which is subsequently wrapped in plastic to prevent water evaporation.
4. Freezing and Measurement: The sample is frozen at a preset temperature for a minimum of 6 hours until complete freezing. The thermal conductivity is then measured in a controlled test environment.
5. Data Collection and Analysis: Each test group consists of at least three parallel samples, yielding a minimum of nine sets of stable data. If significant data dispersion is observed, additional tests are conducted until nine stable data sets are obtained.
6. Data Processing: Abnormal data points are excluded, and the remaining data are averaged. Further averaging is performed for data points within a range of ±3 mean errors. If fewer than five data points fall within this range, data within a ±5% mean error range are selected for averaging and considered as the measured thermal conductivity for the sample group.

## 3. Test results and analysis

### 3.1 Effects of water content on the thermal conductivity of improved soil

**Fig 2:**
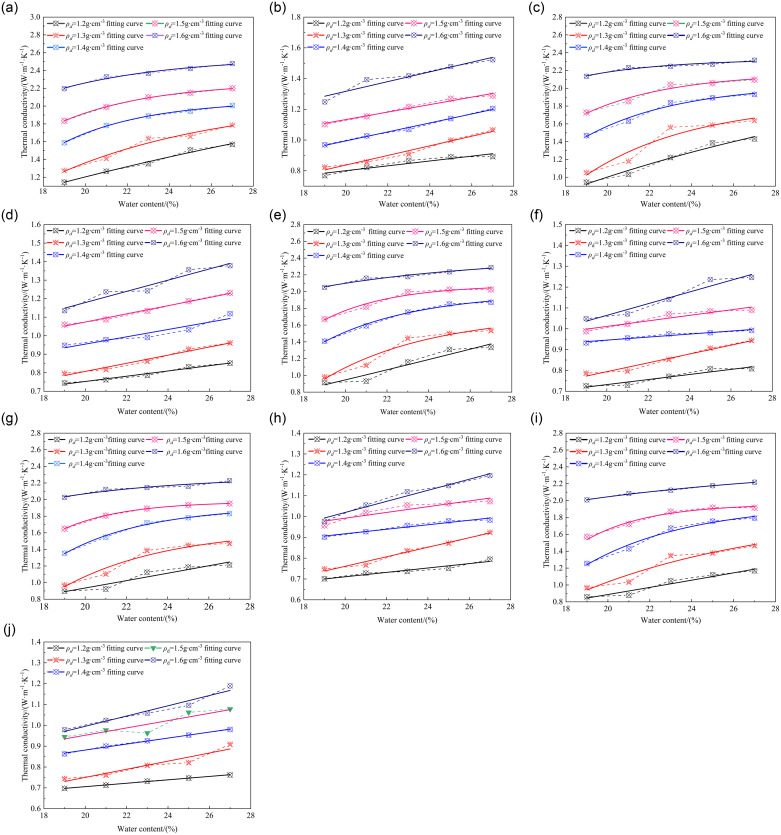
The relationship between thermal conductivity and water content.

The water content of soil exerts a profound influence on its properties, notably its thermal conductivity. Comprising primarily of water and air within pores, variations in water content under constant conditions modify the soil’s three-phase volume fraction, thereby significantly impacting its thermal conductivity. Consequently, a rigorous quantitative analysis of the effect of water content on soil thermal conductivity is paramount. This study, by meticulously manipulating various water content levels, systematically examines the intricate relationship between enhanced soil thermal conductivity and water content across diverse freezing and thawing cycles (in the unfrozen state), as illustrated in Fig 2.

Fig 2 vividly depicts the correlation between the thermal conductivity of improved soil, subjected to varying freeze-thaw cycles, and its water content. The analysis unveils a distinct trend: In the frozen state, the thermal conductivity of the improved soil exhibits an exponential surge with an escalation in water content. In contrast, during the unfrozen state, a linear augmentation in thermal conductivity is discernible as water content rises. To precisely capture these relationships for frozen and thawed soil conditions, regression equations (Eqs (1) and (2), respectively) are presented, offering quantitative insights into the dynamic interplay between soil’s thermal properties and its moisture content.

$$\lambda =a+be^{c\omega }$$ (1)

λ=a+bω (2)

Where *a*, *b*, *c* are parameters, *ω* represents the water content, unit: %. The parameters corresponding to the fitted curve in Fig 2 are shown in Table 3.

**Table 3:** Fitting curve parameter table.

Factor		Parameter				
Dry density/(g∙cm^-3^)	Freeze-thaw cycles/(times)	Freezing state			Unfrozen state	
		*a*	*b*	*c*	*a*	*b*
1.2	1	3.1228	-3.5706	-0.0311	0.0158	0.4844
1.3	1	2.0750	-8.7723	-0.1255	0.0313	0.2111
1.4	1	2.0630	-52.7926	-0.2482	0.0293	0.4093
1.5	1	2.2654	-33.5083	-0.2291	0.0244	0.6462
1.6	1	2.5475	-11.1278	-0.1829	0.0032	0.6839
1.2	3	2.5376	-4.1753	-0.0500	0.0142	0.4688
1.3	3	1.8408	-31.4913	-0.1922	0.0220	0.3682
1.4	3	2.0422	-40.5179	-0.2231	0.0198	0.5596
1.5	3	2.1715	-42.9133	-0.2396	0.0221	0.6329
1.6	3	2.3292	-19.4611	-0.2440	0.0303	0.5739
1.2	5	-6.3124	6.1606	0.0082	0.0122	0.4890
1.3	5	1.7285	-30.2051	-0.1930	0.0212	0.3700
1.4	5	1.9871	-39.3819	-0.2215	0.0073	0.8005
1.5	5	2.0800	-145.2222	-0.3080	0.0133	0.7463
1.6	5	2.4086	-3.7284	-0.1245	0.0281	0.5033
1.2	7	-9.9189	10.0057	0.0041	0.0106	0.5000
1.3	7	1.6432	-29.1943	-0.1968	0.0229	0.3039
1.4	7	1.9193	-54.9500	-0.2403	0.0108	0.7010
1.5	7	1.9755	-193.3715	-0.3359	0.0138	0.7160
1.6	7	2.2797	-4.9677	-0.1583	0.0266	0.4884
1.2	9	-4.4625	4.5755	0.0078	0.0082	0.5416
1.3	9	1.8673	-7.3386	-0.1089	0.0195	0.3601
1.4	9	1.9580	-31.8305	-0.1996	0.0144	0.5944
1.5	9	1.9689	-122.8886	-0.3010	0.0176	0.6014
1.6	9	2.4433	-1.9845	-0.0803	0.0246	0.5034

The thermal conductivity of the improved soil underwent marked variations in response to varying water content and freeze-thaw cycles. Across 1 to 9 freeze-thaw cycles specifically, the thermal conductivity escalated by 27.3%, 33.7%, 33.6%, 30.1%, and 32.5%, respectively, with each incremental increase in water content. Conversely, in the absence of freezing, the soil’s thermal conductivity incremented by 21.7%, 18.1%, 13.4%, 16.1%, and 16.1%, correspondingly. Within the specified ranges of water content, dry density, and freeze-thaw cycles, a discernible trend emerges: the thermal conductivity of the improved soil intensifies concurrently with the augmentation of water content. Notably, this phenomenon is evident in both frozen and unfrozen states, with respective enhancements of 31.4% and 17.1%.

The relationship between the volumetric components of soil, *V*_*V*_, *V*_*ω*_, and *V*_*Air*_, can be expressed as *V*_*V*_ = *V*_*ω*_ + *V*_*Air*_. Under identical conditions, when the volume of voids, *V*_*V*_, remains constant, an increase in water content, *V*_*ω*_, results in a corresponding decrease in air volume, *V*_*Air*_. Given the inherently higher thermal conductivity of water compared to air, the thermal conductivity of soil escalates concomitantly with the increase in water content. Furthermore, during the frozen state, water undergoes a phase transition from its liquid form, characterized by lower thermal conductivity, to solid ice, exhibiting a notably higher thermal conductivity. Consequently, under equivalent conditions, the thermal conductivity of the improved soil in its frozen state surpasses that of the unfrozen improved soil.

### 3.2 Effects of dry density on the thermal conductivity of improved soil

**Fig 3:**
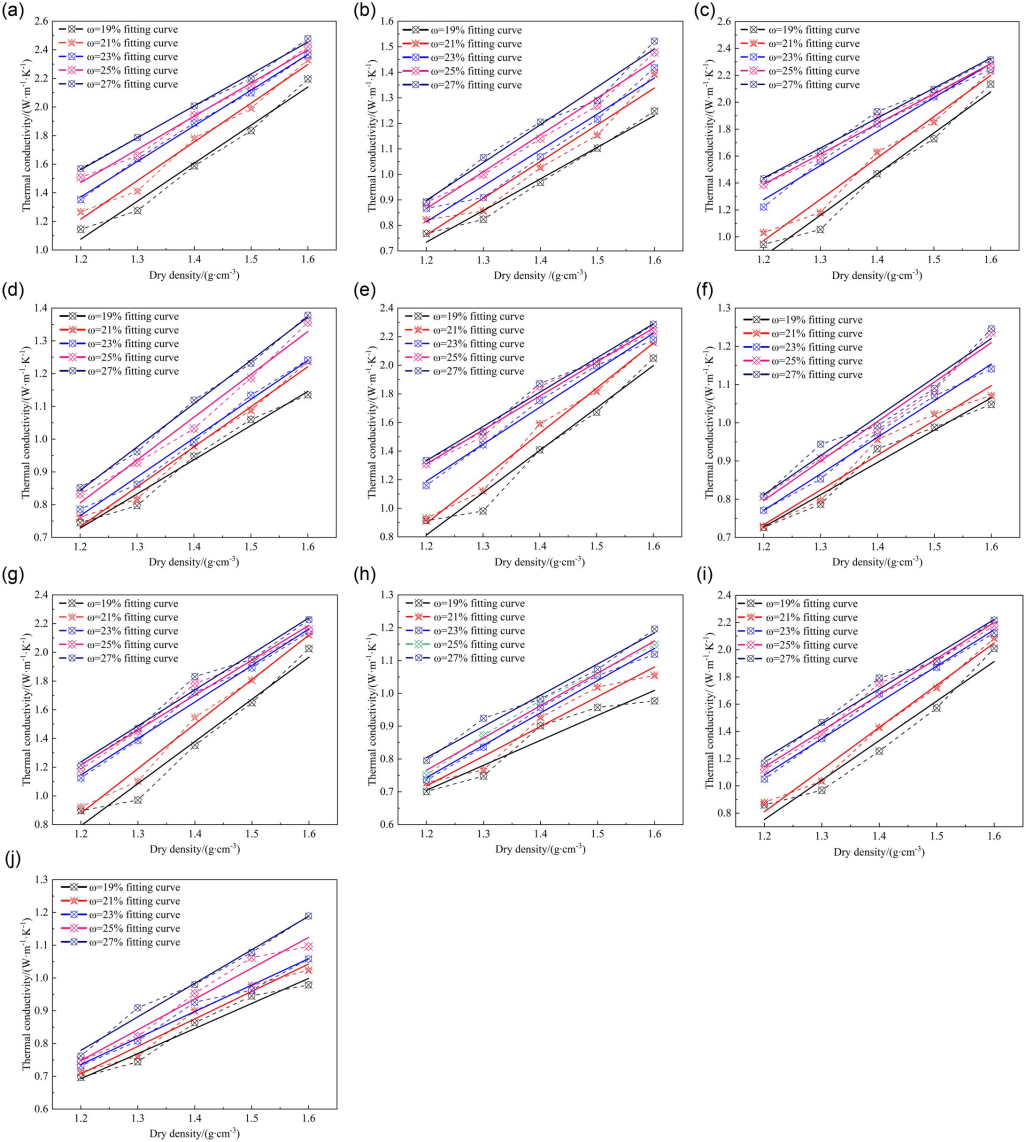
Relationship between thermal conductivity and dry density.

The adjustment of the dry density of the ameliorated soil significantly modulates the interfacial contacts between soil particles and the relative proportions within the soil’s three-phase system. These alterations, in effect, profoundly impact the soil’s thermal conductivity. As elucidated in Fig 3, the relationship between thermal conductivity and dry density underscores the pronounced influence of freeze-thaw cycles on the thermal conductive properties of the improved soil across varying freezing and thawing conditions.

By systematically manipulating variations in water content and freeze-thaw cycles, this study delved into the correlation between the thermal conductivity of the improved soil and its dry density. The findings reveal that, across both frozen and unfrozen soil conditions, within the scope of influencing factors, an augmentation in dry density consistently results in a gradual increase in the thermal conductivity of the improved soil subjected to diverse freeze-thaw cycles. Notably, the relationship between thermal conductivity and dry density exhibits a linear trend, precisely captured by the fitting formula presented in Eq (3).


$$\lambda =a\rho_{d}+b$$ (3)

In the formula, *a* and *b* are parameters. Indicates *ρ*_*d*_ dry density, unit: g∙cm^-3^。The parameters corresponding to the fitting curve in Fig 3 are shown in Table 4.

**Table 4:** Fitting formula parameter table.

Factor		Parameter			
Water content /(%)	Freeze-thaw cycles/(times)	Freezing state		Unfrozen state	
		*a*	*b*	*a*	*b*
19	1	2.6610	-2.1178	1.2390	-0.7522
21	1	2.7050	-2.0306	1.4390	-0.9640
23	1	2.4910	-1.6186	1.4090	-0.8768
25	1	2.3180	-1.3092	1.4460	-0.8692
27	1	2.2310	-1.1152	1.4830	-0.8814
19	3	3.0570	-2.8138	1.0430	-0.5232
21	3	3.0730	-2.7164	1.2210	-0.7328
23	3	2.5300	-1.7602	1.1840	-0.6546
25	3	2.2390	-1.2956	1.3070	-0.7628
27	3	2.2290	-1.2384	1.3220	-0.7426
19	5	2.9650	-2.7462	0.8450	-0.2868
21	5	3.1550	-2.8928	0.9100	-0.3588
23	5	2.5900	-1.9196	0.9570	-0.3778
25	5	2.3770	-1.5434	1.0330	-0.4430
27	5	2.3940	-1.5408	1.0220	-0.4148
19	7	2.9360	-2.7312	0.7610	-0.2086
21	7	3.1010	-2.8412	0.9060	-0.3692
23	7	2.5450	-1.9088	0.9840	-0.4372
25	7	2.4300	-1.6998	0.9850	-0.4164
27	7	2.5080	-1.7726	0.9510	-0.3372
19	9	2.9020	-2.7300	0.7650	-0.2254
21	9	3.0990	-2.9086	0.8380	-0.2982
23	9	2.6680	-2.1222	0.8070	-0.2324
25	9	2.6530	-2.0446	0.9390	-0.3788

As the number of freeze-thaw cycles escalates from 1 to 9, the thermal conductivity of the improved soil in the frozen state undergoes a substantial enhancement within the spectrum of dry density influence, varying from a 73.9% augmentation to a 111.5% surge. Conversely, in the unfrozen state, the thermal conductivity exhibits a declining trend, with an initial 66.5% increase at the first freeze-thaw cycle diminishing to a 46.2% increment after nine cycles. Incremental enhancements in dry density by 0.1 g∙cm^-3^ correspond to commensurate increases in the thermal conductivity of the modified soil, amounting to 0.263 W·m⁻¹·K⁻¹ under frozen conditions and 0.106 W·m⁻¹·K⁻¹under unfrozen conditions, respectively. This underscores the profound sensitivity of thermal conductivity to variations in dry density, irrespective of the soil’s frozen state.

A meticulous analysis reveals that the escalation of dry density facilitates a gradual augmentation in the thermal conductivity of the improved soil. Notably, as dry density intensifies, the influence of freeze-thaw cycles on the thermal conductivity of frozen improved soil attenuates. Furthermore, elevated dry densities decelerate the development of freeze-thaw-induced damage and pore cracking, leading to a mitigated reduction in soil thermal conductivity. Consequently, as the count of freeze-thaw cycles proliferates, the thermal conductivity of the improved soil progressively augments. In contrast, for unfrozen improved soil, the intensification of freeze-thaw cycles becomes pronounced. Despite the augmentation in dry density, the thermal conductivity of unfrozen improved soil gradually deteriorates with an increase in freeze-thaw cycles, attributable to the absence of filling and ice cementation effects that arise from the volumetric expansion during the water-ice phase transition within soil pores, as compared to frozen soil.

### 3.3 Effects of freeze-thaw cycles on the thermal conductivity of improved soil

**Fig 4:**
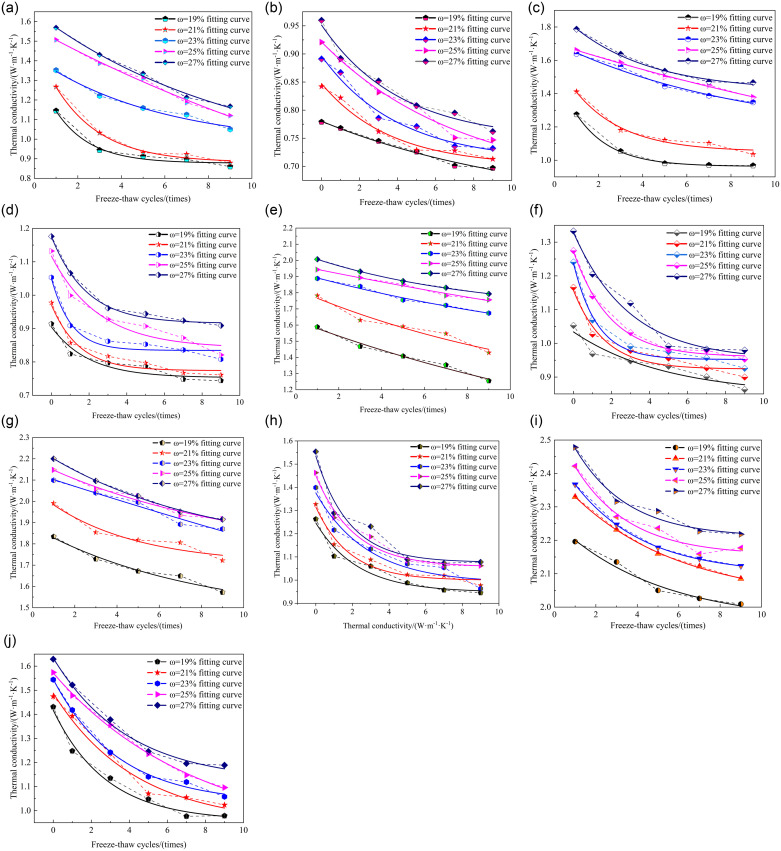
The relationship between the thermal conductivity of the improved soil and the freeze-thaw cycle.

This study delves into the freeze-thaw cycle, a natural process predominantly driven by the cyclic transformation of water between liquid and solid phases, meticulously examining how it reshapes soil structure and porosity, thereby exerting a pervasive influence on various soil properties. To precisely quantify the specific impact of freeze-thaw cycles on soil thermal conductivity, a systematic investigation was conducted, varying the number of freeze-thaw cycles as the primary variable. Fig 4 visually depicts the dynamic relationship between soil thermal conductivity and the number of freeze-thaw cycles.

As Fig 4 illustrates, a profound trend emerges from the comparative analysis of soil thermal conductivity in both frozen and unfrozen states across varying freeze-thaw cycles: an exponential decline in thermal conductivity with the increasing number of cycles. To further substantiate this observation, a fitting formula (4) is introduced, elegantly capturing the functional relationship between soil thermal conductivity in both frozen and thawed states as a function of water content. This formula provides a robust theoretical foundation for understanding the mechanisms of soil heat transfer under freeze-thaw action.


$$\lambda =a+be^{cD}$$ (4)

In the formula: *a*, *b*, *c* are parameters. *D* represents the number of freeze-thaw cycles, unit: time. The parameters corresponding to the fitted curve in Fig 4 are shown in Table 5.

**Table 5:** Fitting curve parameters.

Factor		Parameter					
Dry density/(g∙cm^-3^)	Water content/(%)	Freezing state			Unfrozen state		
		*a*	*b*	*c*	*a*	*b*	*c*
1.2	19	0.8775	0.4983	-0.6271	0.6222	0.1581	-0.0883
1.2	21	0.8814	0.6182	-0.4725	0.7009	0.1465	-0.2775
1.2	23	0.9700	0.4476	-0.1735	0.7095	0.1869	-0.2506
1.2	25	0.2251	1.3402	-0.0454	0.6658	0.2556	-0.1352
1.2	27	0.8618	0.7892	-0.1081	0.7478	0.2037	-0.2428
1.3	19	0.9614	0.5897	-0.6358	0.7530	0.1520	-0.5028
1.3	21	1.0518	0.5610	-0.4521	0.7741	0.1969	-0.6779
1.3	23	1.1432	0.5639	-0.1162	0.8347	0.2161	-0.9604
1.3	25	0.6233	1.0789	-0.0394	0.8416	0.2757	-0.3950
1.3	27	1.4168	0.4947	-0.2828	0.9155	0.2598	-0.5427
1.4	19	0.6710	0.9571	-0.0528	0.8477	0.1862	-0.2048
1.4	21	1.0876	0.7338	-0.0784	0.9238	0.2333	-0.6111
1.4	23	1.3616	0.5668	-0.5663	0.9523	0.2852	-0.8019
1.4	25	0.9071	1.0648	-0.0259	0.9600	0.3124	-0.5166
1.4	27	1.6790	0.3735	-0.1307	0.9515	0.3773	-0.3402
1.5	19	1.4547	0.4259	-0.1312	0.9480	0.2986	-0.9438
1.5	21	1.7054	0.3528	-0.2391	0.9991	0.3165	-0.5414
1.5	23	-0.0873	2.2221	-0.0147	0.9879	0.3882	-0.3547
1.5	25	1.7324	0.4603	-0.1053	1.0565	0.3940	-0.4766
1.5	27	1.7584	0.5040	-0.1320	1.0766	0.4582	-0.5465
1.6	19	1.9545	0.3009	-0.2012	0.9582	0.4588	-0.3577
1.6	21	2.0210	0.3751	-0.1935	0.9436	0.5444	-0.2322
1.6	23	2.0993	0.3618	-0.3007	1.0328	0.5124	-0.2943
1.6	25	2.1555	0.3878	-0.3806	0.8802	0.6910	-0.1316
1.6	27	2.2097	0.3937	-0.3940	1.1222	0.5117	-0.2550

For the improved soil in the frozen state, within the range of 1 to 9 freeze-thaw cycles, an inverse correlation is discernible between dry density and the decrement in thermal conductivity with an increasing number of cycles. Specifically, as the dry density ascends from 1.2 g∙cm^-3^ to 1.6 g∙cm^-3^, the reduction in thermal conductivity diminishes from 25.8% to 10.0%. Conversely, in the case of unfrozen improved soil, the decrement in thermal conductivity exhibits a direct proportionality to the number of freeze-thaw cycles. Across the spectrum of dry densities, this decrement escalates from 16.6% at 1.2 g∙cm^-3^ to 30.2% at 1.6 g∙cm^-3^.

A notable observation is that the influence of freeze-thaw cycles on the thermal conductivity of stabilized soil in a frozen state is less pronounced compared to its counterpart in the thawed state. An augmentation in dry density acts as a mitigating factor, alleviating the impact of freeze-thaw cycles on soil thermal conductivity. Conversely, elevated water content exacerbates the influence of these cycles on the thermal conductivity of stabilized soil. Thus, the intricate interplay among water content, dry density, and freeze-thaw cycles collectively orchestrates the variation in soil thermal conductivity.

### 3.4 Analysis and establishment of empirical model

#### 3.4.1 Analysis

**Fig 5:**
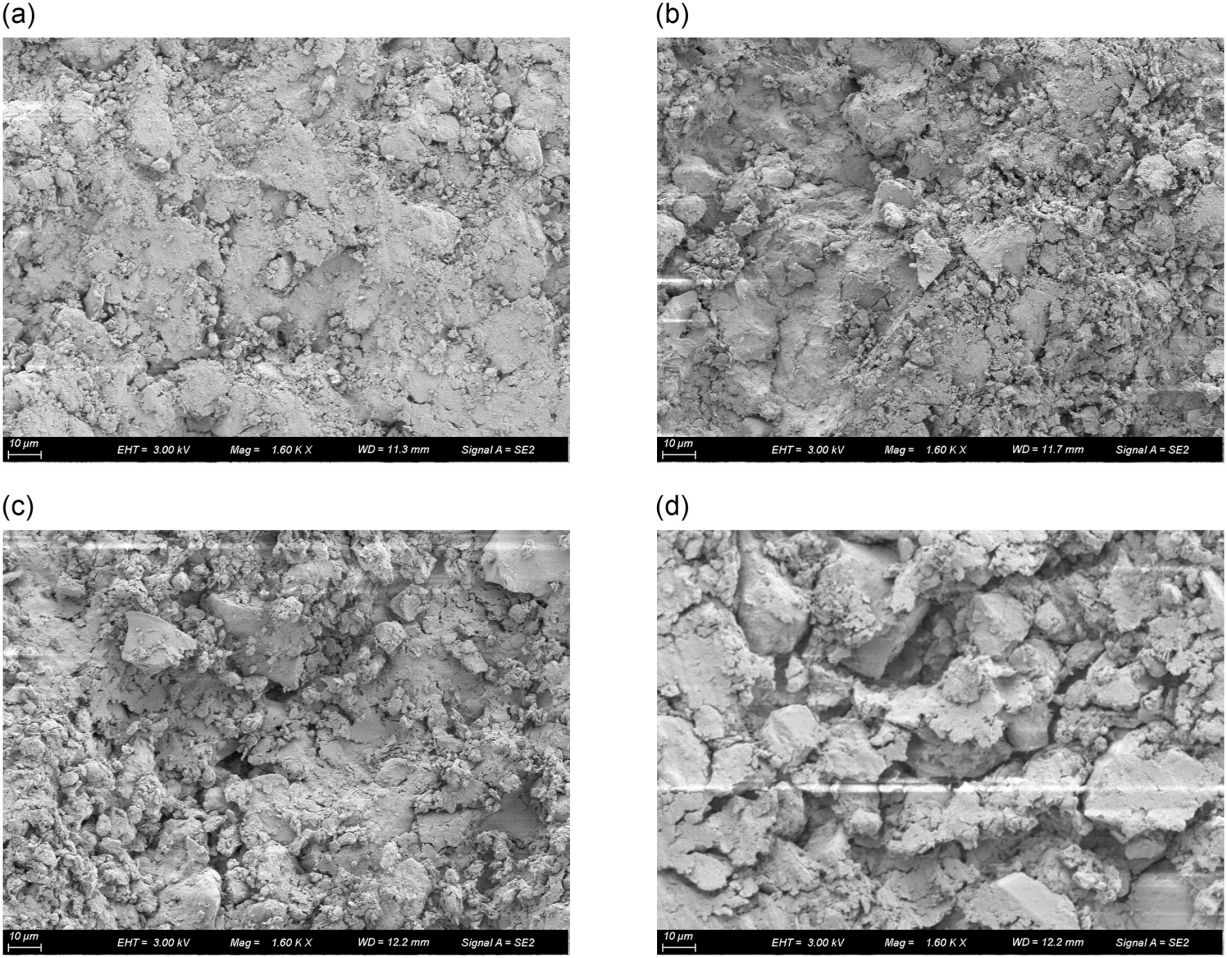
SEM images of improved soil under different freeze-thaw cycles.

As water content escalates, the ice content within frozen soil correspondingly rises. During soil freezing, the phase transition from water to ice induces a volumetric expansion of approximately 1/9, thereby diminishing pore volume within unsaturated frozen soil. The augmented thermal conductivity of frozen soil with higher water content is primarily ascribed to the diminished internal soil pores and the enhanced thermal conductivity associated with the phase change of water to ice. Conversely, in unfrozen soil, an increase in water content diminishes soil air volume, gradually elevating thermal conductivity. An enhancement in dry density corresponds to an improvement in soil compactness, where a heightened soil density translates into a larger particle contact area, subsequently expanding the effective heat transfer zone and enhancing thermal conductivity. The scanning electron microscopy (SEM) images in Fig 5 illustrate the microstructural evolution of soil post various freeze-thaw cycles.

**Fig 6:**
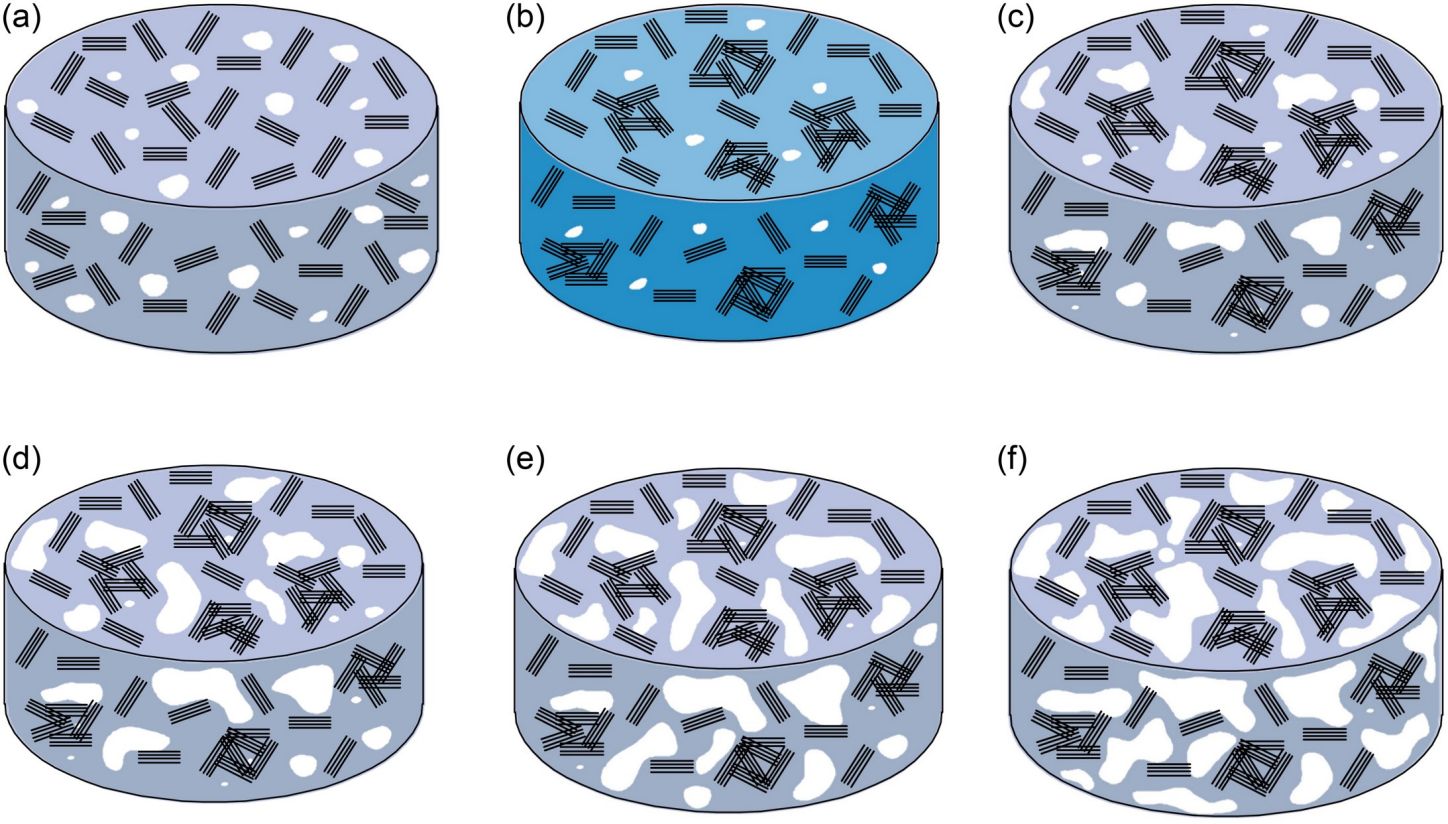
The schematic diagram of the change law of the internal pores of the sample during the freeze-thaw cycle. Note: The white area in the figure is used to indicate the area occupied by pores, cracks or air parts, and the black area indicates soil particles.

Fig 5 showcases the SEM images of improved soil subjected to different freeze-thaw cycles. An augmentation in the number of freeze-thaw cycles leads to a progressive enlargement of pores and cracks within the soil matrix, accompanied by an increase in their volumes, subsequently altering the volumetric proportions of the soil’s three-phase components. The expansion of air volume results in a gradual decrement in soil thermal conductivity. Fig 6 presents a schematic representation of the internal pore variation pattern within the sample during the freeze-thaw cycle.

Fig 6(a) to 6(c) depict the sample undergoing a complete freeze-thaw cycle, encompassing the transition from preparation to freezing and subsequent thawing during the test. Fig 6(d) to 6(f) illustrate the internal soil structure in the thawed state after 3, 5, and 9 freeze-thaw cycles, respectively. In the initial state, the soil exhibits a uniform dispersion of particles, water, and pores due to artificial preparation. Upon freezing, water within the sample transforms into ice, causing a volumetric expansion of approximately 1/9, reducing pore volume, decreasing the uniformity of soil particle dispersion, and enhancing local compactness. Concurrently, the thermal conductivity of frozen soil surpasses that of unfrozen soil due to ice’s higher thermal conductivity compared to water. Upon melting, the ice phase reverts to water, leading to an increase in pores and cracks, a decrease in saturation, and a reduction in soil thermal conductivity compared to the initial state. As the number of freeze-thaw cycles accumulates, the repeated phase change between ice and water augments the volume and count of soil pores and fissures, gradually diminishing the soil’s thermal conductivity.

#### 3.4.2 The establishment of empirical model

**Fig 7:**
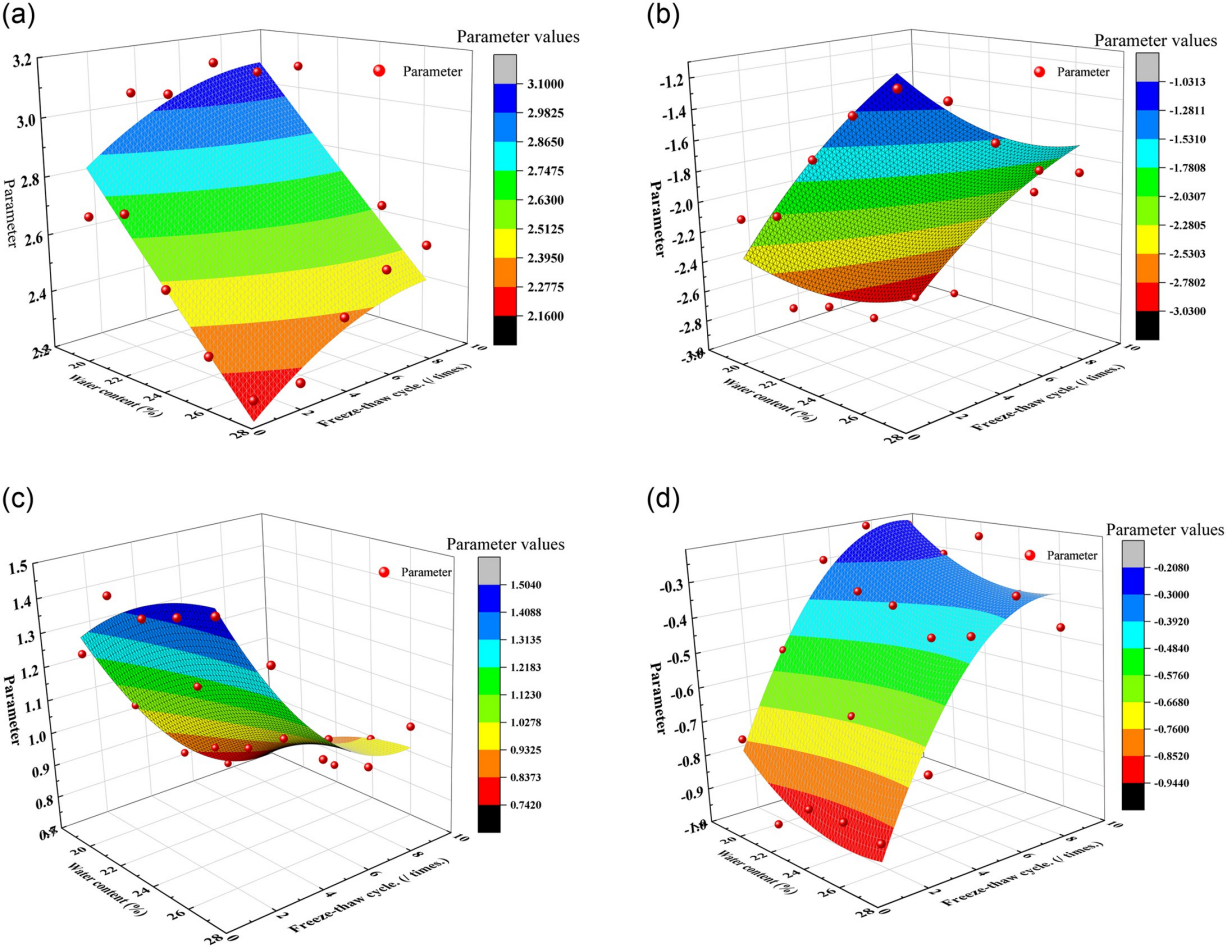
Empirical model establishment process.

To precisely quantify the impact of water content, dry density, and freeze-thaw cycles on the thermal conductivity of lime-modified red clay, this study delves into the evolution of thermal conductivity under the combined effects of these three factors. By meticulously comparing and analyzing fitting formulas (1) through (4) and their corresponding curve parameters detailed in Tables 3 to 5, formula (3) and Table 4 were selected for model development, owing to their simplicity and minimal parameter set. Fig 7 illustrates the intricate empirical modeling process in detail.

During the modeling phase, a three-dimensional scatter plot was constructed, followed by a surface fitting analysis that comprehensively integrated water content, the number of freeze-thaw cycles, and the fitting parameters pertaining to the thermal conductivity and dry density of the modified soil. This exhaustive analysis culminated in the derivation of a fitting formula as presented in Eq (5), offering a quantitative framework for understanding the individual and combined effects of the factors on thermal conductivity.

$$y={\text{y}}_{0}+\text{a}\omega +\text{bD}+\text{c}\omega^{2}{+\text{dD}}^{2}$$ (5)

where *a*, *b*, *c* are parameters, *ω* represents the water content, unit: %; *D* represents the number of freeze-thaw cycles, unit: times. The fitting surface parameters shown in Fig 7 are shown in Table 6.

**Table 6:** Fitting surface parameters.

Fig number	Fig 7(a)	Fig 7(b)	Fig 7(c)	Fig 7(d)
Parameter				
y_0_	4.5223	-8.3721	-0.5323	1.4214
a	-0.0990	0.4331	0.1578	-0.2001
b	0.0713	-0.1654	-0.1602	0.1761
c	0.0003	-0.0058	-0.0028	0.0040
d	-0.0038	0.0084	0.0092	-0.0104

Substitute the formula (5) into the formula (3), and obtain the empirical formula model as shown in formula (6):

$$\left\{\begin{array}{l} y=A\rho_{d}+C\\ A={\text{y}}_{1}+{\text{a}}_{1}\omega +{\text{b}}_{1}{\text{D}+\text{c}}_{1}\omega^{2}+{\text{d}}_{1}{\text{D}}^{2}\\ C={\text{y}}_{2}+{\text{a}}_{2}\omega +{\text{b}}_{2}\text{D}+{\text{c}}_{2}\omega^{2}+{\text{d}}_{2}{\text{D}}^{2} \end{array}\right.$$ (6)

In the formula, *A*, *C*, *a*_*1*_, *b*_*1*_, *c*_*1*_ *y*_*1*_, *a*_*2*_, *b*_*2*,_ *c*_*2*_, *y*_*2*_ are all parameters, *ω* represents the water content, unit: %; *D* represents the number of freeze-thaw cycles, unit: times; *ρ*_*d*_ is expressed as dry density, unit: g∙cm^-3^.

During the freeze-thaw cycle, when the soil undergoes improvement and reaches a frozen state, the parameters *A* and *C* within the formula are specified in Eq (7).


$$\left\{\begin{array}{l} A=4.5223-0.0990\omega +0.0713\text{D}+0.0003\omega^{2}-0{.0038\text{D}}^{2}\\ C=-8.3721+0.4331\omega -0.1654\text{D}-0.0058\omega^{2}+0.0084{\text{D}}^{2} \end{array}\right.$$ (7)

During the freeze-thaw cycle, when the improved soil remains in an unfrozen state, the pertinent parameters in the formula are presented in Formula (8).:

$$\left\{\begin{array}{l} A=-0.5323+0.1578\omega -0.1602\text{D}-0.0028\omega^{2}+0{.0092\text{D}}^{2}\\ C=1.4214-0.20001\omega +0.1761D+0.0040\omega^{2}-0.0104{\text{D}}^{2} \end{array}\right.$$ (8)

#### 3.4.3 Validation analysis of empirical model

**Fig 8:**
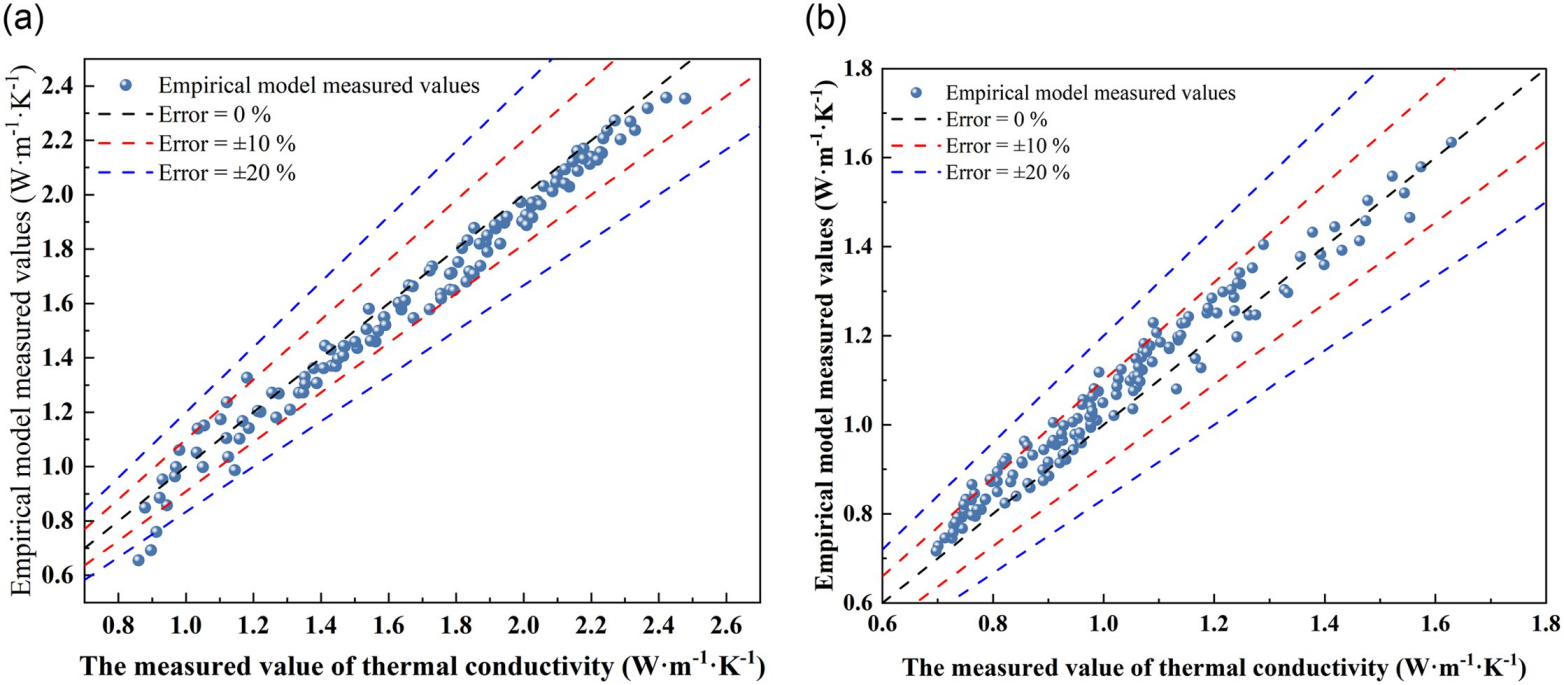
Comparison of measured and predicted values of empirical model prediction.

A predictive model was devised to capture the evolution of thermal conductivity in lime-modified red clay, accounting for the intricate interplay of water content, dry density, and freeze-thaw cycles. To rigorously substantiate its validity, the empirical model (6) was harnessed to forecast the thermal conductivity of the improved soil, with a comparison diagram (Fig 8) showcasing the measured versus predicted values.

**Fig 9:**
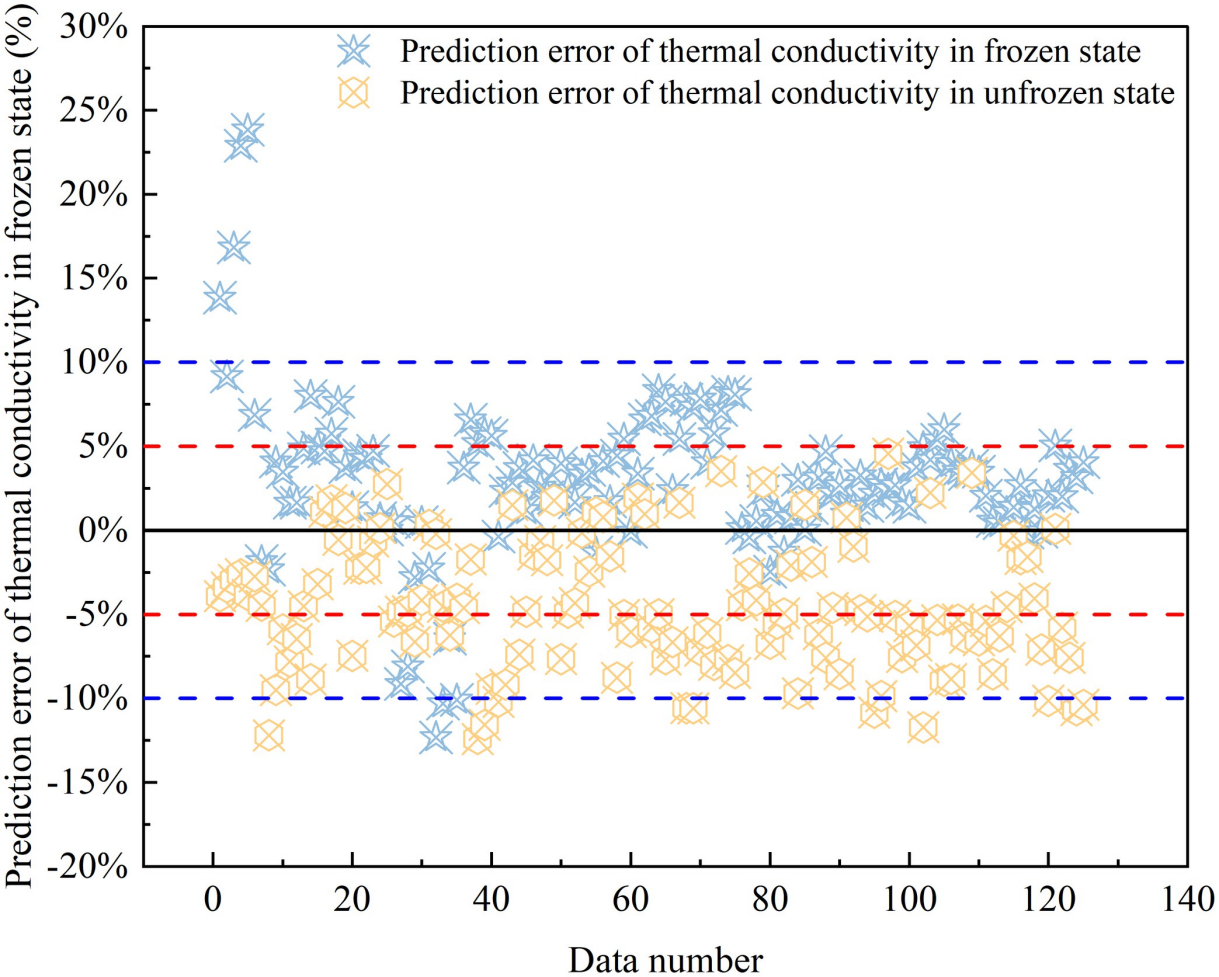
Empirical model prediction error analysis.

Fig 8 presents a visual juxtaposition of the empirical model’s predicted and measured thermal conductivity values. The model demonstrates commendable predictive prowess, yielding satisfactory results for both frozen and unfrozen conditions of the two improved soils. Notably, the prediction errors primarily cluster within a 10% margin, with slightly elevated predictions for frozen soil and marginally lower ones for unfrozen soil. To further gauge the model’s precision, an empirical model prediction error analysis chart (Fig 9) is presented.

Fig 9 delves into the prediction errors of the improved soil’s thermal conductivity under both frozen and unfrozen states. For frozen soil, 94.4% of predictions exhibit errors below 10%, with 72.8% falling within a tighter 5% margin. In the unfrozen state, 90% of predictions maintain errors under 10%, with a maximum error of 13.62% and a minimal error of 0.08%. The root mean square errors (RMSE) for the predicted thermal conductivity of the improved soil are 0.0771 and 0.0637, respectively, for frozen and unfrozen conditions. The average absolute error percentages stand at 4.17% and 5.62%, correspondingly, for the frozen and unfrozen states. Notably, the empirical model underscores its high predictive accuracy, enabling quantitative insights into the thermal conductivity of lime-modified red clay, while comprehensively considering the coupled effects of multiple factors.

## 4. Construction and application of set model

### 4.1 The construction of BP neural network

The Backpropagation (BP) neural network, a type of feedforward neural network, fundamentally comprises an input layer, hidden layers, and an output layer [15]. It stands as one of the most prevalent neural network models in various applications [16, 17]. In regression prediction contexts, the input layer of the BP neural network encapsulates the crucial factors influencing the prediction outcomes, while the output layer represents the predicted results [18].

**Fig 10:**
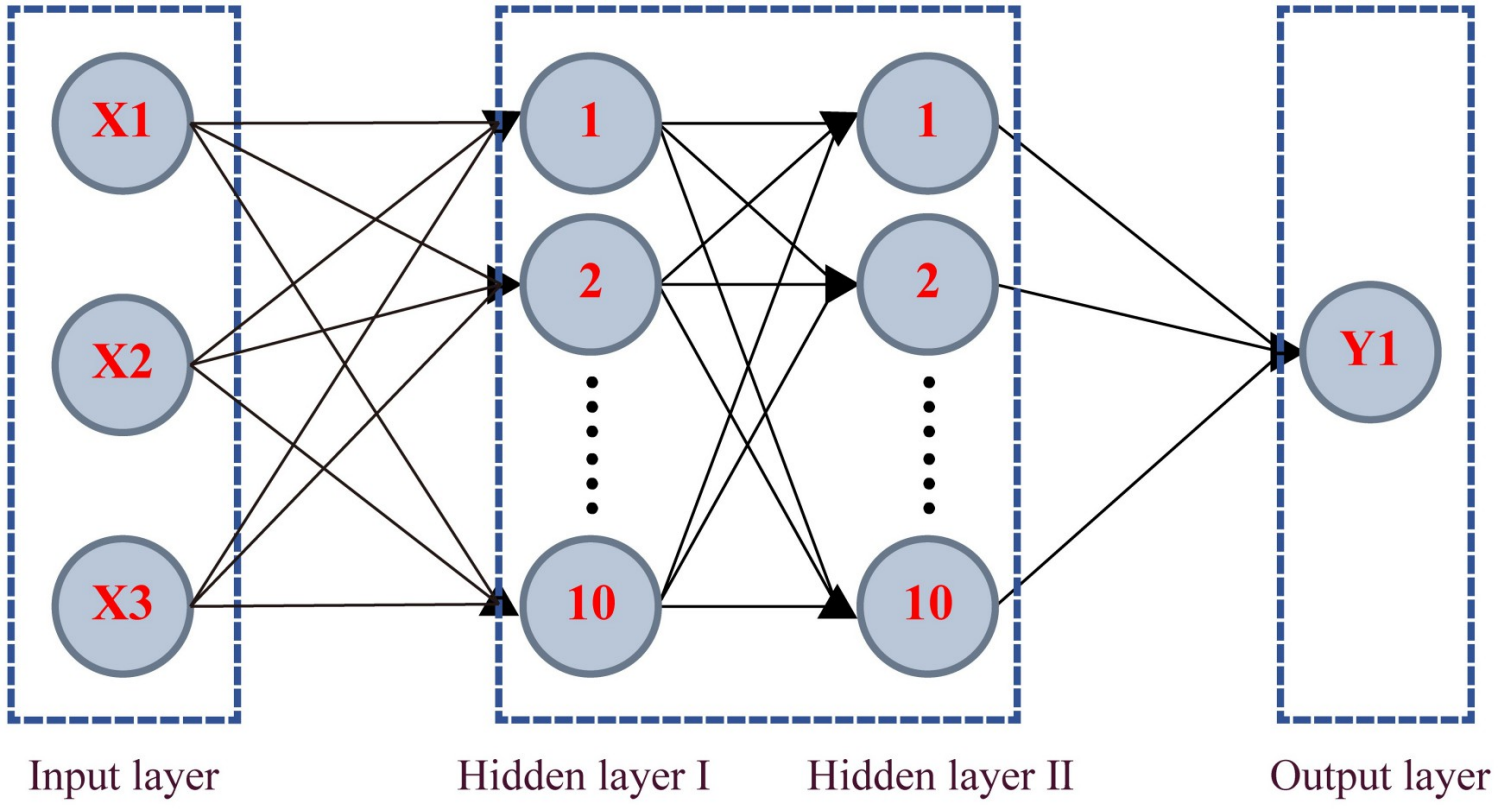
BP neural network structure diagram.

The thermal conductivity of the enhanced soil under investigation is influenced by multiple factors, including water content, dry density, and freeze-thaw cycles. Consequently, the input layer of the BP neural network primarily consists of these three factors, each represented by a distinct neuron. The output layer, on the other hand, encodes the thermal conductivity, represented by a single neuron. The network architecture incorporates two hidden layers, each housing 10 neurons, as depicted in Fig 10.

The construction process of the BP neural network adheres to the following steps:

1. Data Acquisition and Preparation: Initially, data is organized in an Excel spreadsheet, with each column labeled for clarity and ease of analysis.
2. Dataset Partitioning: The dataset is partitioned into training and testing subsets, with the latter comprising 20% of the total data.
3. Data Normalization: The data is standardized using the StandardScaler function from the sklearn library to ensure consistent scaling.
4. Network Architecture and Parameterization: The BP neural network model is defined with a three-tiered structure, where each hidden layer contains 10 neurons, optimized through experimentation. Key parameters, such as the activation function, optimizer, and loss function, are meticulously configured for optimal prediction performance.
5. Activation Function Selection: Among ReLU, Sigmoid, and tanh, tanh is chosen due to its ability to maintain gradient flow and mitigate gradient vanishing issues, making it suitable for deep neural networks.
6. Optimizer Selection: The RMSProp optimizer, with its attenuation factor, is selected to ensure efficient training by addressing gradient attenuation during optimization.
7. Loss Function Determination: Given the regression nature of the study, the MSELoss function is adopted as it emphasizes larger deviations, suitable for predicting continuous values.
8. Model Training and Preservation: The model is trained based on the defined parameters, with 500 iterations to ensure convergence. To prevent overfitting, the training data is shuffled before each epoch. Upon completion, the trained BP neural network model is preserved for further validation and analysis.

### 4.2 The construction of linear regression model

In this study, to account for the intricate interplay among water content, dry density, and freeze-thaw cycles, we employ a multivariate linear regression model (LR-M) that is adept at capturing nonlinearities, enabling accurate forecasting of the thermal conductivity of the enhanced soil. This model comprehensively characterizes these three influential factors, associating each sample data point with a predicted thermal conductivity value and a D-dimensional vector W, where D specifically equals 3 in this context. Linear regression models, broadly speaking, encompass generalized formulations that span from univariate linear regression to their derivative counterparts, such as logistic regression and ridge regression [19]. Consequently, the predicted value for a given sample is formulated according to Eq (9).

$$y_{i}=b+\sum_{d=1}^{D}w_{d}x_{i,d}$$ (9)

Where *b* = *w*_*D*_+1, *x*_*i*,*d*_, represent the i-th eigenvalue in the d-th sample.

By equivalent substitution, the parameters in Formula (9) are eliminated as shown in Formula (10).


$$y_{i}=\sum_{d=1}^{D+1}w_{d}x_{i,d}$$ (10)

The dimensionality of the original *D*-vector is augmented to *D*+1, with the updated vector configuration detailed in Eqs (11) through (13).


$$W=\left[\begin{array}{c} w_{1}\\ w_{2}\\ \begin{array}{c} \vdots \\ w_{D} \end{array}\\ w_{D+1} \end{array}\right]$$ (11)

$$X=\left[\begin{array}{c} x_{1}\\ x_{2}\\ \vdots \\ x_{N} \end{array}\right]=\left(\begin{array}{ccccc} x_{1,1} & x_{1,2} & \cdots & x_{1,D} & 1\\ x_{2,1} & x_{2,2} & \cdots & x_{2,D} & 1\\ \vdots & \vdots & \ddots & \vdots & \vdots \\ x_{N,1} & x_{N,2} & \cdots & x_{N,D} & 1 \end{array}\right)$$ (12)

$$y=\left[\begin{array}{c} y_{1}\\ y_{2}\\ \vdots \\ y_{N} \end{array}\right]$$ (13)

Further available:

$$y_{i}=\sum_{d=1}^{D+1}w_{d}x_{i,d}=W^{T}X_{i}$$ (14)

That is:

y=XW (15)

In the multiple linear regression model, the loss function of the model is shown in (16):

$$\text{L}({\hat{\text{y}}}_{i}{,y}_{i})={\left({\hat{\text{y}}}_{i}-{\text{y}}_{i}\right)}^{2}$$ (16)

### 4.3 The construction of GBDT model

**Fig 11:**
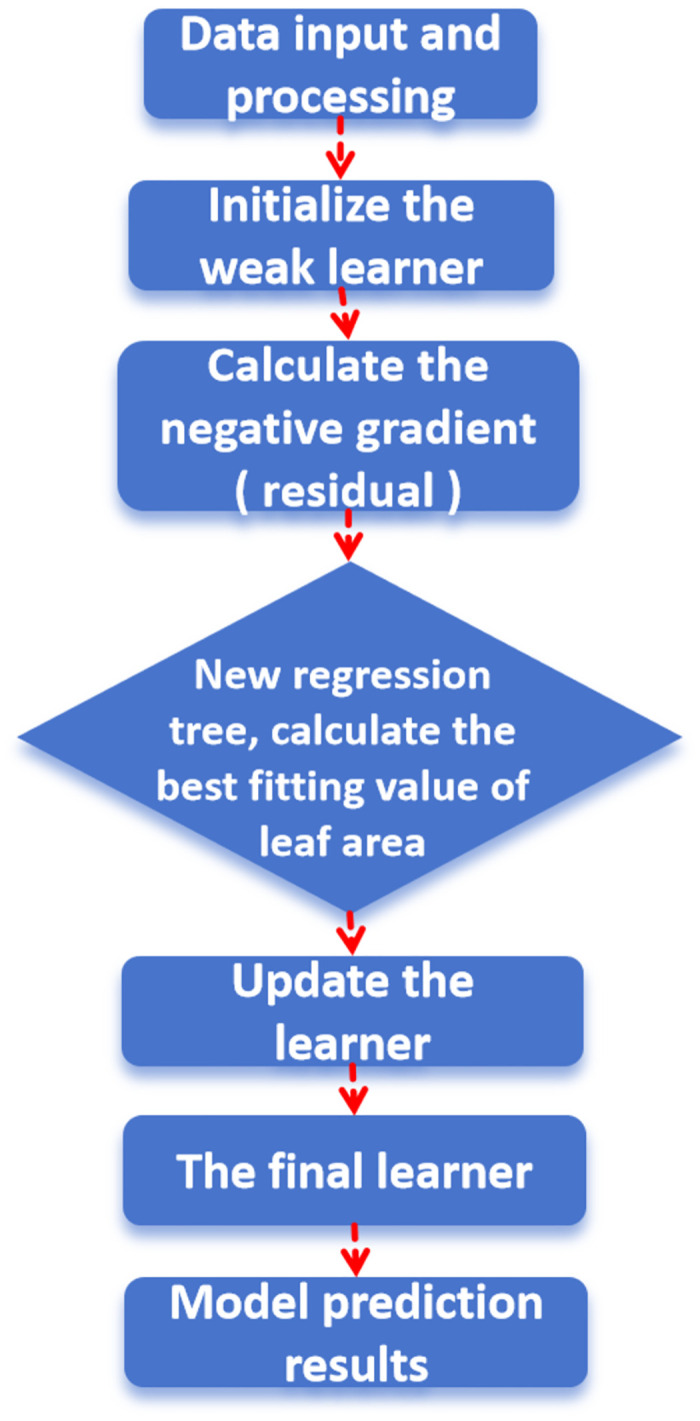
Structure diagram of GBDT model.

In this research, the Gradient Boosting Decision Tree (GBDT) model, an advanced iteration of the Classification and Regression Tree (CART) algorithm, is employed. As reported by Ilyas et al. [20], GBDT constructs a series of CARTs and aggregates their outputs to achieve comprehensive predictions. Drawing upon our investigation into the thermal conductivity of improved soil, a GBDT model has been formulated, with its structural diagram presented in Fig 11.

The model’s construction process encompasses the following critical steps:

1. Data Processing and Set Division: The process of data reading and subsequent division rigorously adheres to the standards established for the previously utilized BP neural network model, ensuring data consistency and comparability.
2. Model Definition and Tree Parameter Setting: The model’s definition is meticulously finalized using the sklearn framework. To optimize performance, the staged_predict() method is leveraged to automatically search for the optimal number of training trees, determined through rigorous error analysis.
3. Other Parameter Configurations: Following extensive error analysis of the model, the learning rate is empirically set to 0.01 through a trial-and-error approach. The maximum tree depth is fixed at 500, while all remaining parameters are maintained at their default values to preserve model stability and efficiency.

### 4.4 The construction of the ensemble model

To elevate the predictive performance and achieve high-precision forecasting, a comprehensive approach integrating BP Neural Network (BPNN), Gradient Boosting Decision Tree (GBDT), and Logistic Regression-Mixed model (LR-M) is proposed. By parallelizing these three individual models and conducting a comparative analysis of their prediction effects using absolute error as the metric, the predicted value that best aligns with the measured value is determined by selecting the one with the minimal absolute error between predictions and measurements. This ensemble methodology aspires to construct a diverse ensemble of machine learning models, thereby enhancing prediction accuracy while mitigating issues such as gradient vanishing, exploding gradients, low prediction accuracy, intricate model parameter tuning, and data sensitivity that are pervasive in standalone models.

**Fig 12:**
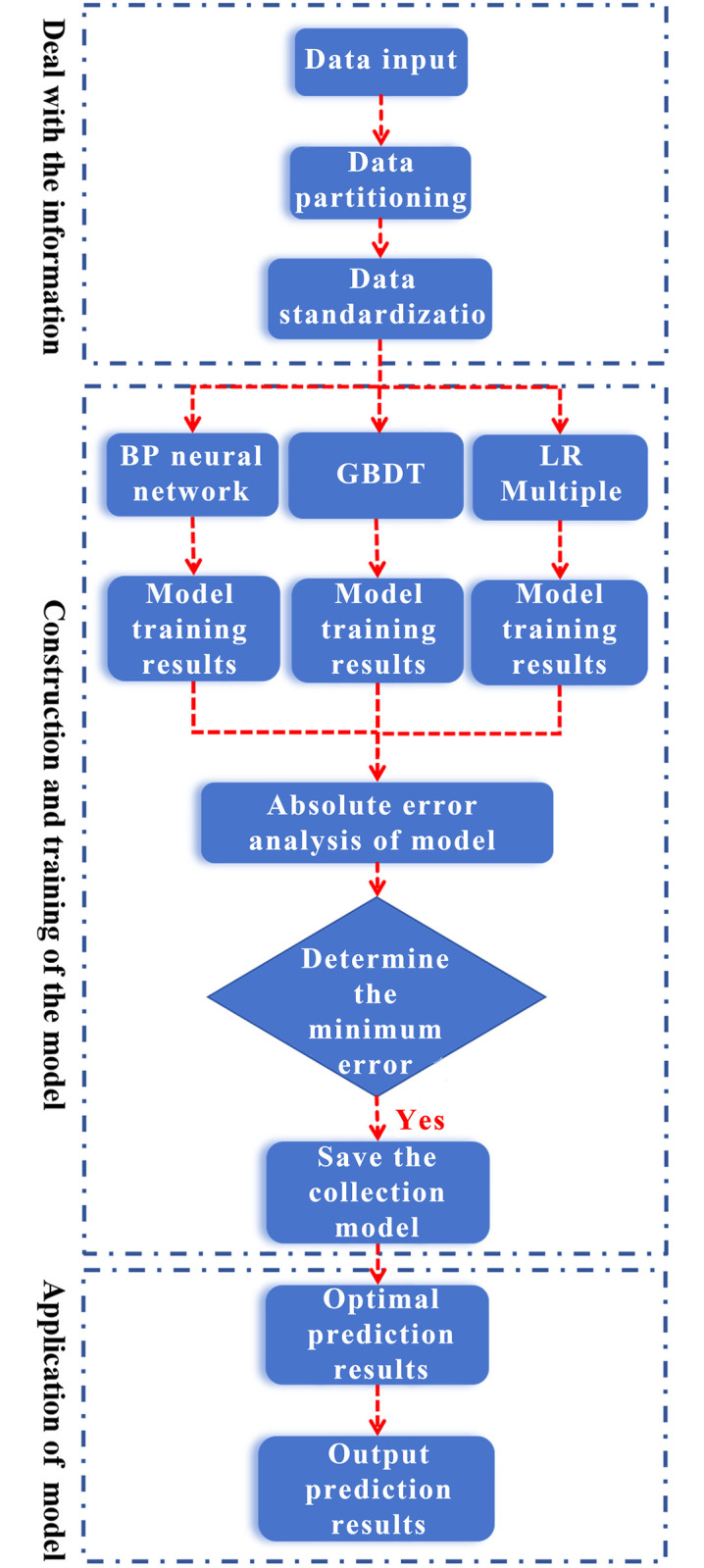
Structure diagram of ensemble model.

To maintain consistency and enable an effective comparison, the predictive capabilities of both the individual models and the ensemble model (E-M) are evaluated side by side. In the ensemble model, the structural configurations and parameter settings of BPNN, GBDT, and LR-M remain unaltered. Fig 12 showcases the schematic diagram of the ensemble model.

### 4.5 Application analysis of model

**Fig 13:**
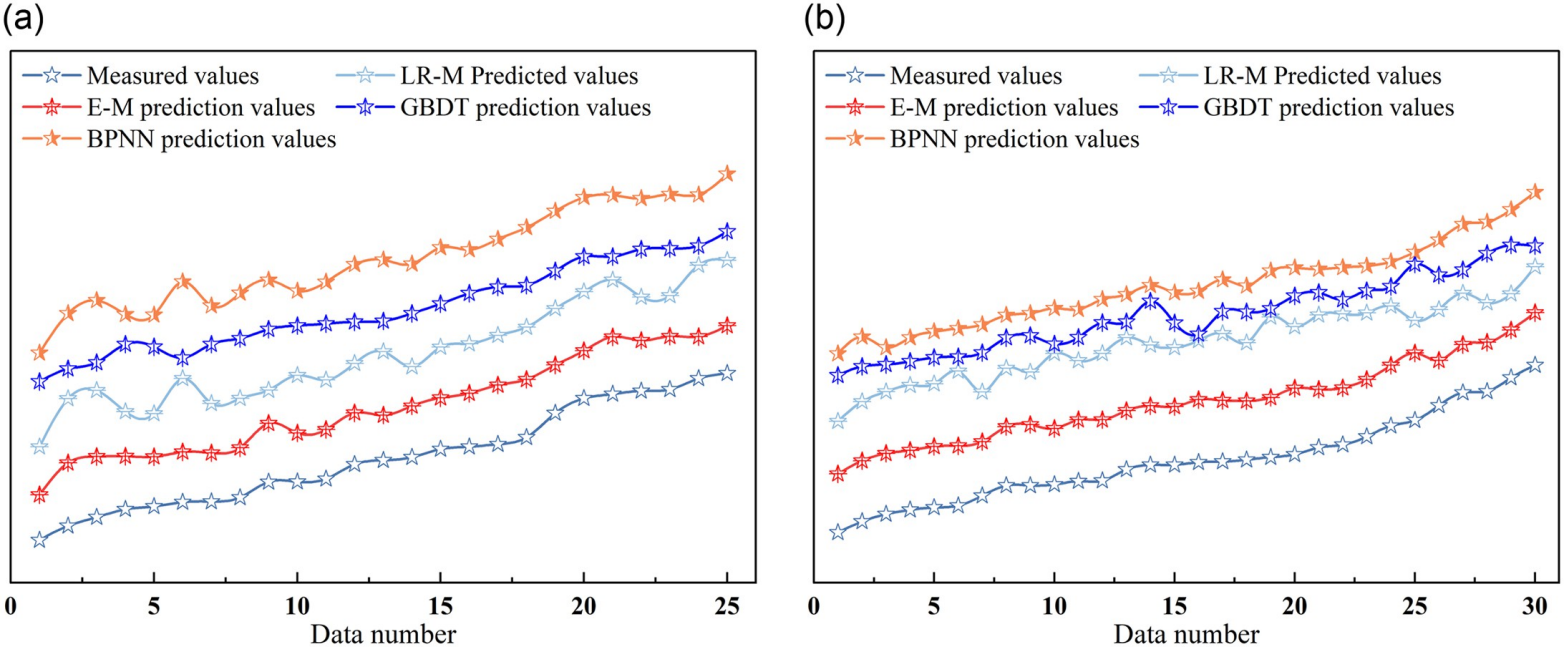
Model prediction error trend chart.

This study employed three individual models and their integrated model (E-M) to predict and analyze the thermal conductivity of lime-modified red clay subjected to freeze-thaw cycles. Subsequently, a model prediction error trend chart was constructed (as shown in Fig 13), offering profound insights into the accuracy and reliability of the various models.

In Fig 13(a), a comparative analysis of predicted data from different models against the corresponding measured dataset is presented. For frozen soil, the predicted values from the GBDT and E-M models closely follow the measured values, indicating high correlation. For thawed soil, Fig 13(b) demonstrates that the BPNN and E-M models exhibit the most precise predictions of thermal conductivity in the improved soil, with predicted trends mirroring the measured values.

**Fig 14:**
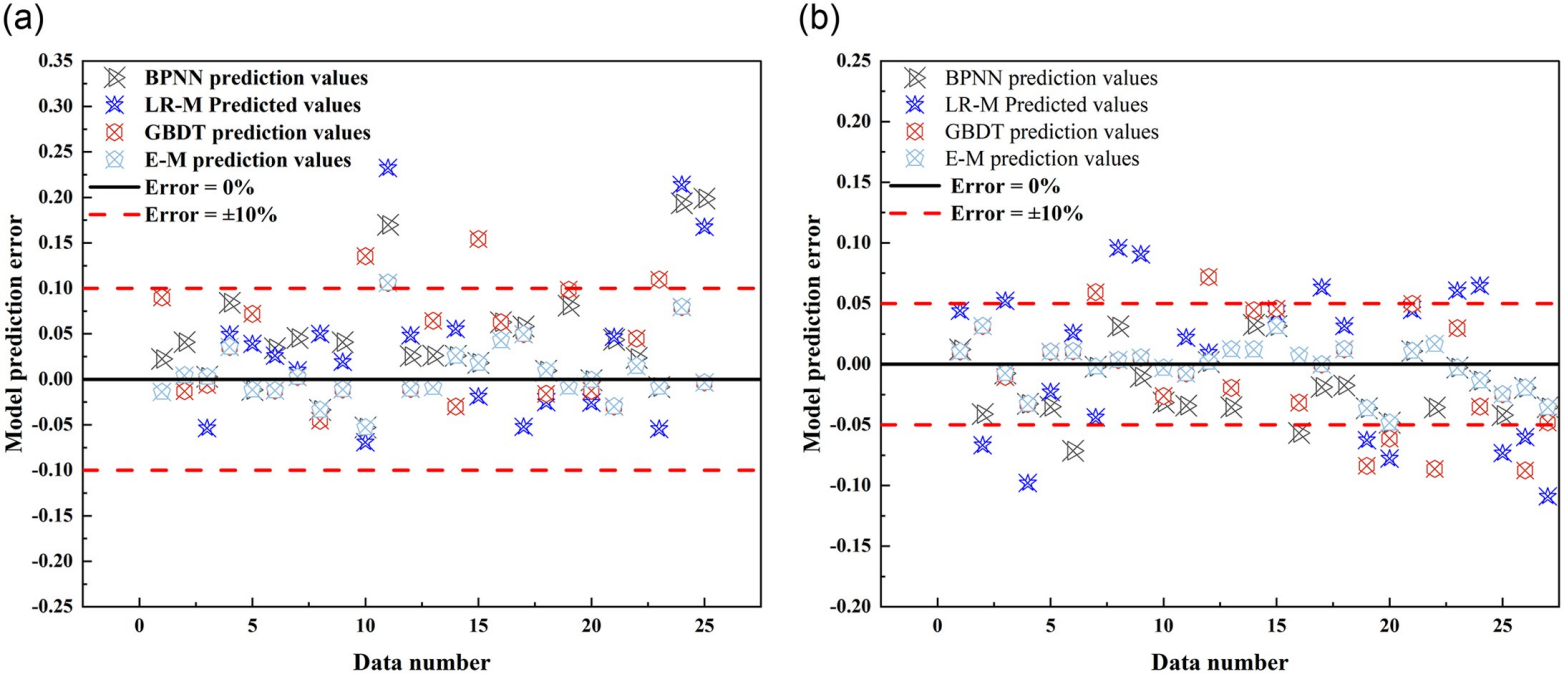
Model prediction error analysis.

To delve deeper into the predictive capabilities of individual and ensemble models regarding the thermal conductivity of improved soil, a model prediction error analysis diagram is presented (as shown in Fig 14).

Fig 14(a) and 14(b) showcase the error analysis of predicted thermal conductivity values for the improved soil using various models. Notably, the E-M model exhibits the highest prediction accuracy for frozen soil, with 96% of predictions having an error margin below 10% and 88% within a 5% error range. The maximum prediction error is 10.60%, while the minimum prediction error is as low as 0.09%. For thawed soil, the E-M model also demonstrates optimal predictive performance, with all predictions having an error below 5%. Specifically, the maximum prediction error is 4.83%, and the minimum prediction error is merely 0.02%.

After rigorous analysis, it becomes evident that the ensemble model, constructed by integrating three individual models, significantly enhances prediction accuracy. To comprehensively evaluate the performance of the E-M model, key metrics such as Root Mean Square Error (RMSE) and Mean Absolute Percentage Error (MAPE) were calculated for the BPNN, GBDT, LR-M, and E-M models. The results, tabulated in Table 7 below, provide a quantitative assessment of the effectiveness of the ensemble approach.

**Table 7:** Model prediction error analysis index.

Model	Soil state	RMSE	MAPE
BPNN	Freezing state	0.093	5.26%
	Unfrozen state	0.036	2.90%
LR-M	Freezing state	0.096	5.40%
	Unfrozen state	0.062	4.28%
GBDT	Freezing state	0.081	5.18%
	Unfrozen state	0.053	3.40%
E-M	Freezing state	0.044	2.39%
	Unfrozen state	0.023	1.29%

By scrutinizing the RMSE and MAPE values across diverse models, the superior predictive prowess of the ensemble model (E-M) becomes evident, underscoring the potential of ensemble methods in bolstering the reliability and robustness of predictive modeling. As exhibited in Table 7, the BPNN, GBDT, LR-M, and E-M models all yielded satisfactory predictions for the thermal conductivity of lime-modified red clay, considering influential factors such as water content, dry density, and freeze-thaw cycles. Among the fundamental single models, GBDT exhibited a heightened suitability for predicting frozen soil’s thermal conductivity, while BPNN outperformed in thawed soil predictions. Nonetheless, the E-M model, with its superlative prediction accuracy and reduced error indices, comprehensively surpassed the three individual models.

Specifically, in predicting the thermal conductivity of frozen soil, the E-M model demonstrated remarkable predictive capabilities for improved soil, accommodating various factors. Compared to the commendable GBDT model, the E-M model achieved a substantial reduction of 45.68% in RMSE and 53.86% in MAPE. Similarly, for thawing soil, the E-M model surpassed BPNN, notching a notable decrease of 36.11% in RMSE and 55.52% in MAPE. This integration of base models within the E-M framework facilitates precise predictions of soil thermal conductivity, accounting for diverse influences. This advancement underscores the potential of ensemble methods in refining predictive modeling’s dependability and resilience.

**Fig 15:**
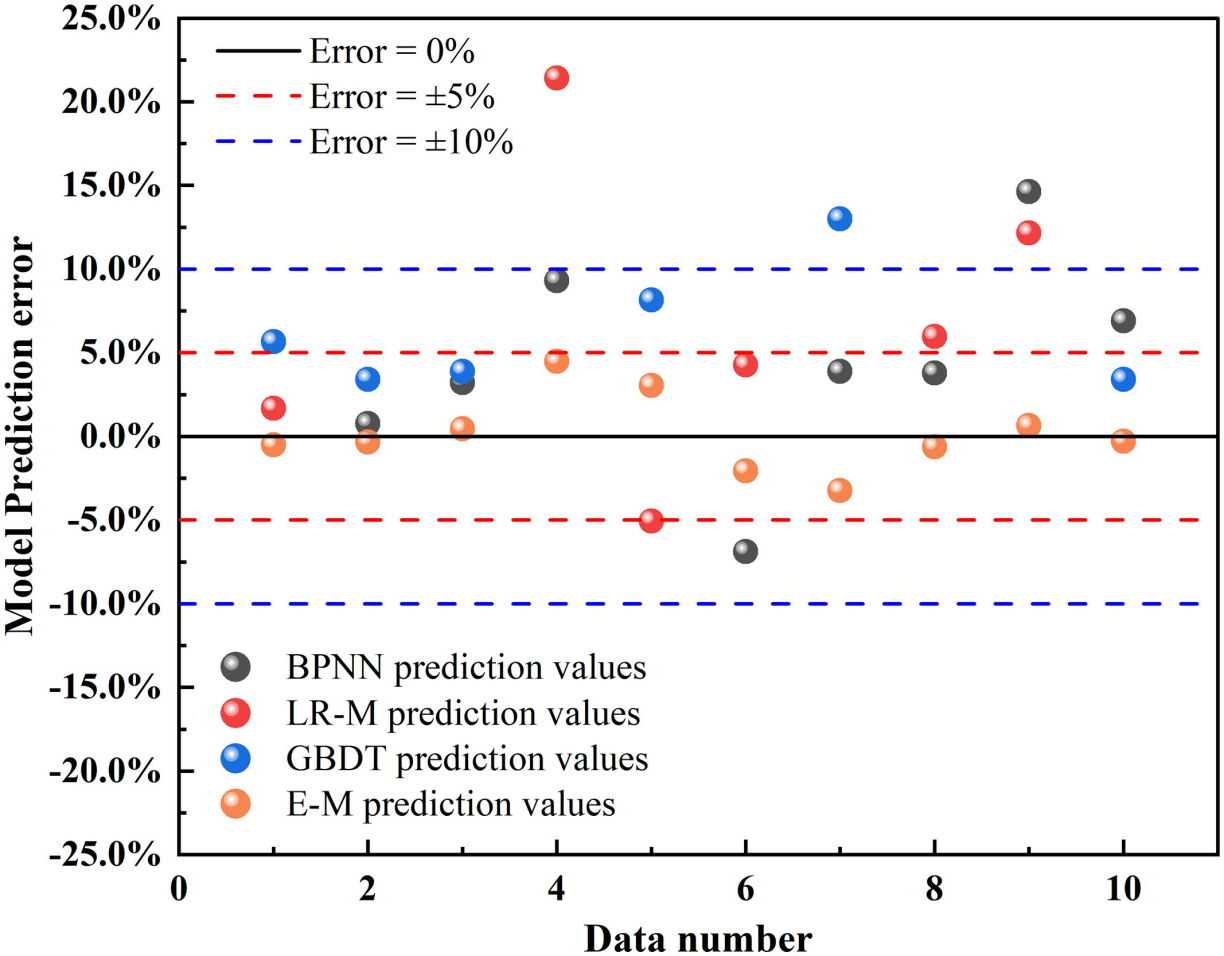
Model generalisation validation error analysis.

To further ascertain the ensemble model’s universality, we validated its validity and generalizability using thermal conductivity coefficients of primary pulverized clay in a frozen state sourced from other scholars’ work [21]. Employing 39 data points while maintaining the model’s relevant parameters unchanged, we conducted a predictive analysis of the thermal conductivity of primary frozen soil under varying dry densities, moisture contents, and temperatures. As illustrated in Fig 15, the ensemble model’s prediction errors are below 5%, with a maximum error of 4.47% and a minimum of 0.35%. In contrast, the maximum prediction errors for BPNN, linear regression, and GBDT were 14.63%, 21.41%, and 13%, respectively, with minimum errors of 0.52%, 0.35%, and 0.63%, respectively. The RMSE and MAPE values for the ensemble model’s predictions were calculated as 0.024 and 1.57%, respectively, demonstrating its paramount prediction accuracy. Consequently, the ensemble model remains a precise predictor even when applied to the thermal conductivity of other clay types.

## 5. Discussion

Despite conducting a comprehensive three-factor, five-level full factorial experiment to investigate the effects of freeze-thaw cycles, moisture content, and dry density on the thermal conductivity of lime-stabilized soil, this study acknowledges several limitations that may influence the generalizability and precision of the findings.

1. Geographic and Material Specificity: Given the variability in properties and mineral composition of red clay across different climatic and geological regions, the conclusions drawn from this study using a specific regional clay may lack universal applicability. Future research should encompass samples from multiple locations and delve deeper into the influence of mineral composition on the thermal performance of modified soil, thereby enhancing the comprehensiveness and generalizability of the results.
2. Challenges in Sample Preparation and Uniformity: The limited quantity of lime used and the small size of test specimens pose challenges in achieving uniform mixing and minimizing inter-batch variability. To mitigate these issues, we recommend adopting a one-time, large-scale sample preparation approach and increasing the number of parallel samples (to at least 5–6 sets) to ensure sample homogeneity and avoid the need for supplemental samples, which may introduce further uncertainties in uniformity.
3. Data Dispersion and Measurement Precision: The occurrence of high data dispersion, particularly after numerous freeze-thaw cycles due to altered pore structures, necessitates enhanced measurement precision. Adjusting sample interfaces and conducting a larger number of replicate tests are suggested to accumulate more concentrated data points, facilitating robust data processing and analysis, and ultimately leading to more accurate conclusions.
4. Overlooked Acid-Base Interaction: This study focused on the effects under freeze-thaw and low-temperature conditions, overlooking the temporal evolution of acid-base interactions between lime and red clay at ambient temperatures. Further detailed investigations into these interactions under low-temperature conditions are warranted to fully comprehend the long-term performance of lime-stabilized soil across varying environments.

## 6. Conclusion

This study delves into the influence of water content, dry density, freeze-thaw cycles, and other variables on the thermal conductivity of lime-modified red clay, resulting in the development of an empirical model, a standalone machine learning model, and an ensemble model (E-M). The core findings are succinctly encapsulated as follows:

1. Within the examined parameters, both water content and dry density exhibit positive correlations with the thermal conductivity of the improved soil. Specifically, an increase in water content augments the thermal conductivity by 31.44% and 17.08% in frozen and unfrozen states, respectively. Likewise, an average rise of 0.1 g∙cm^-3^ in dry density corresponds to an enhancement of 0.263 and 0.106 W·m^-1^·K^-1^ in thermal conductivity for frozen and unfrozen conditions.
2. Conversely, the freeze-thaw cycle adversely affects thermal conductivity, with an exponential decrease observed as the number of cycles increases. Notably, the magnitude of this reduction gradually diminishes with further increments in cycle count.
3. Rooted in the analysis of the thermal conductivity of the improved soil under the combined influence of three factors, empirical and E-M models were formulated. The E-M model integrates BPNN, GBDT, and LR-M approaches. Comparative assessments reveal that all models proficiently predict the thermal conductivity under varying conditions. Secondary validation using experimental data from other researchers confirms the models’ remarkable prediction accuracy, with the E-M model based on a single machine learning algorithm emerging as the most precise, significantly bolstering predictive efficacy. These insights offer invaluable perspectives for the examination of temperature fields in improved soils and the research and forecasting of thermal conductivity in comparable soil samples.

## Supporting information

S1 File (DOCX)
