# Supplementary material for: Quantitative investigation and intelligent forecasting of thermal conductivity in lime-modified red clay
Source: PLoS One. 2024 Oct 10;19(10):e0311882. doi: 10.1371/journal.pone.0311882 (PMC11469617; doi:10.1371/journal.pone.0311882)
Supplement: S1 File — (DOCX) [file pone.0311882.s001.docx]

Data availability statement

In this paper, all the measured data are plotted as point and line diagrams (Figs 2-4). All the data of point-line diagrams are from real measurement data, which contains 275 real measurement data in total.

In this study, the formula model is mainly based on the evolution law of the original data. The machine learning models are all constructed based on Python. The relevant model codes can be obtained by contacting the first author, Hongqi Wang (Emil : 1979483119 @ qq.com). The authors will send emails upon request.

The following table (S1 Table Measured Data) shows the 275 raw data obtained experimentally in this study. (S2 Table Cited Data) shows the data from others that were cited in the secondary validation of the model during this study.

**S1 Table Measured data**

| Water content /(%) | Dry density /(g·cm−3) | Freeze-thaw cycle /(times) | State of soil sample | Thermal conductivity /(W·m-1·K-1) |
| --- | --- | --- | --- | --- |
|  |  |  |  |  |
| 19 | 1.2 | 1 | Freezing state. | 1.145 |
| 19 | 1.2 | 3 | Freezing state. | 0.944 |
| 19 | 1.2 | 5 | Freezing state. | 0.913 |
| 19 | 1.2 | 7 | Freezing state. | 0.897 |
| 19 | 1.2 | 9 | Freezing state. | 0.86 |
| 19 | 1.3 | 1 | Freezing state. | 1.275 |
| 19 | 1.3 | 3 | Freezing state. | 1.054 |
| 19 | 1.3 | 5 | Freezing state. | 0.981 |
| 19 | 1.3 | 7 | Freezing state. | 0.971 |
| 19 | 1.3 | 9 | Freezing state. | 0.968 |
| 19 | 1.4 | 1 | Freezing state. | 1.588 |
| 19 | 1.4 | 3 | Freezing state. | 1.468 |
| 19 | 1.4 | 5 | Freezing state. | 1.408 |
| 19 | 1.4 | 7 | Freezing state. | 1.353 |
| 19 | 1.4 | 9 | Freezing state. | 1.255 |
| 19 | 1.5 | 1 | Freezing state. | 1.834 |
| 19 | 1.5 | 3 | Freezing state. | 1.729 |
| 19 | 1.5 | 5 | Freezing state. | 1.672 |
| 19 | 1.5 | 7 | Freezing state. | 1.649 |
| 19 | 1.5 | 9 | Freezing state. | 1.572 |
| 19 | 1.6 | 1 | Freezing state. | 2.196 |
| 19 | 1.6 | 3 | Freezing state. | 2.135 |
| 19 | 1.6 | 5 | Freezing state. | 2.05 |
| 19 | 1.6 | 7 | Freezing state. | 2.026 |
| 19 | 1.6 | 9 | Freezing state. | 2.009 |
| 21 | 1.2 | 1 | Freezing state. | 1.267 |
| 21 | 1.2 | 3 | Freezing state. | 1.032 |
| 21 | 1.2 | 5 | Freezing state. | 0.931 |
| 21 | 1.2 | 7 | Freezing state. | 0.923 |
| 21 | 1.2 | 9 | Freezing state. | 0.879 |
| 21 | 1.3 | 1 | Freezing state. | 1.412 |
| 21 | 1.3 | 3 | Freezing state. | 1.181 |
| 21 | 1.3 | 5 | Freezing state. | 1.121 |
| 21 | 1.3 | 7 | Freezing state. | 1.103 |
| 21 | 1.3 | 9 | Freezing state. | 1.035 |
| 21 | 1.4 | 1 | Freezing state. | 1.782 |
| 21 | 1.4 | 3 | Freezing state. | 1.63 |
| 21 | 1.4 | 5 | Freezing state. | 1.591 |
| 21 | 1.4 | 7 | Freezing state. | 1.547 |
| 21 | 1.4 | 9 | Freezing state. | 1.429 |
| 21 | 1.5 | 1 | Freezing state. | 1.991 |
| 21 | 1.5 | 3 | Freezing state. | 1.854 |
| 21 | 1.5 | 5 | Freezing state. | 1.818 |
| 21 | 1.5 | 7 | Freezing state. | 1.806 |
| 21 | 1.5 | 9 | Freezing state. | 1.722 |
| 21 | 1.6 | 1 | Freezing state. | 2.33 |
| 21 | 1.6 | 3 | Freezing state. | 2.232 |
| 21 | 1.6 | 5 | Freezing state. | 2.16 |
| 21 | 1.6 | 7 | Freezing state. | 2.122 |
| 21 | 1.6 | 9 | Freezing state. | 2.085 |
| 23 | 1.2 | 1 | Freezing state. | 1.352 |
| 23 | 1.2 | 3 | Freezing state. | 1.221 |
| 23 | 1.2 | 5 | Freezing state. | 1.159 |
| 23 | 1.2 | 7 | Freezing state. | 1.125 |
| 23 | 1.2 | 9 | Freezing state. | 1.05 |
| 23 | 1.3 | 1 | Freezing state. | 1.638 |
| 23 | 1.3 | 3 | Freezing state. | 1.562 |
| 23 | 1.3 | 5 | Freezing state. | 1.444 |
| 23 | 1.3 | 7 | Freezing state. | 1.387 |
| 23 | 1.3 | 9 | Freezing state. | 1.348 |
| 23 | 1.4 | 1 | Freezing state. | 1.888 |
| 23 | 1.4 | 3 | Freezing state. | 1.839 |
| 23 | 1.4 | 5 | Freezing state. | 1.755 |
| 23 | 1.4 | 7 | Freezing state. | 1.722 |
| 23 | 1.4 | 9 | Freezing state. | 1.674 |
| 23 | 1.5 | 1 | Freezing state. | 2.099 |
| 23 | 1.5 | 3 | Freezing state. | 2.04 |
| 23 | 1.5 | 5 | Freezing state. | 1.996 |
| 23 | 1.5 | 7 | Freezing state. | 1.892 |
| 23 | 1.5 | 9 | Freezing state. | 1.87 |
| 23 | 1.6 | 1 | Freezing state. | 2.367 |
| 23 | 1.6 | 3 | Freezing state. | 2.247 |
| 23 | 1.6 | 5 | Freezing state. | 2.178 |
| 23 | 1.6 | 7 | Freezing state. | 2.145 |
| 23 | 1.6 | 9 | Freezing state. | 2.123 |
| 25 | 1.2 | 1 | Freezing state. | 1.507 |
| 25 | 1.2 | 3 | Freezing state. | 1.387 |
| 25 | 1.2 | 5 | Freezing state. | 1.309 |
| 25 | 1.2 | 7 | Freezing state. | 1.186 |
| 25 | 1.2 | 9 | Freezing state. | 1.12 |
| 25 | 1.3 | 1 | Freezing state. | 1.66 |
| 25 | 1.3 | 3 | Freezing state. | 1.586 |
| 25 | 1.3 | 5 | Freezing state. | 1.501 |
| 25 | 1.3 | 7 | Freezing state. | 1.45 |
| 25 | 1.3 | 9 | Freezing state. | 1.378 |
| 25 | 1.4 | 1 | Freezing state. | 1.943 |
| 25 | 1.4 | 3 | Freezing state. | 1.893 |
| 25 | 1.4 | 5 | Freezing state. | 1.852 |
| 25 | 1.4 | 7 | Freezing state. | 1.782 |
| 25 | 1.4 | 9 | Freezing state. | 1.756 |
| 25 | 1.5 | 1 | Freezing state. | 2.148 |
| 25 | 1.5 | 3 | Freezing state. | 2.059 |
| 25 | 1.5 | 5 | Freezing state. | 2.024 |
| 25 | 1.5 | 7 | Freezing state. | 1.934 |
| 25 | 1.5 | 9 | Freezing state. | 1.917 |
| 25 | 1.6 | 1 | Freezing state. | 2.422 |
| 25 | 1.6 | 3 | Freezing state. | 2.27 |
| 25 | 1.6 | 5 | Freezing state. | 2.236 |
| 25 | 1.6 | 7 | Freezing state. | 2.159 |
| 25 | 1.6 | 9 | Freezing state. | 2.177 |
| 27 | 1.2 | 1 | Freezing state. | 1.569 |
| 27 | 1.2 | 3 | Freezing state. | 1.431 |
| 27 | 1.2 | 5 | Freezing state. | 1.335 |
| 27 | 1.2 | 7 | Freezing state. | 1.213 |
| 27 | 1.2 | 9 | Freezing state. | 1.168 |
| 27 | 1.3 | 1 | Freezing state. | 1.787 |
| 27 | 1.3 | 3 | Freezing state. | 1.637 |
| 27 | 1.3 | 5 | Freezing state. | 1.535 |
| 27 | 1.3 | 7 | Freezing state. | 1.471 |
| 27 | 1.3 | 9 | Freezing state. | 1.466 |
| 27 | 1.4 | 1 | Freezing state. | 2.007 |
| 27 | 1.4 | 3 | Freezing state. | 1.931 |
| 27 | 1.4 | 5 | Freezing state. | 1.872 |
| 27 | 1.4 | 7 | Freezing state. | 1.831 |
| 27 | 1.4 | 9 | Freezing state. | 1.793 |
| 27 | 1.5 | 1 | Freezing state. | 2.2 |
| 27 | 1.5 | 3 | Freezing state. | 2.096 |
| 27 | 1.5 | 5 | Freezing state. | 2.025 |
| 27 | 1.5 | 7 | Freezing state. | 1.951 |
| 27 | 1.5 | 9 | Freezing state. | 1.915 |
| 27 | 1.6 | 1 | Freezing state. | 2.478 |
| 27 | 1.6 | 3 | Freezing state. | 2.316 |
| 27 | 1.6 | 5 | Freezing state. | 2.287 |
| 27 | 1.6 | 7 | Freezing state. | 2.227 |
| 27 | 1.6 | 9 | Freezing state. | 2.218 |
| 19 | 1.2 | 0 | Unfrozen state. | 0.7787 |
| 19 | 1.2 | 1 | Unfrozen state. | 0.9139 |
| 19 | 1.2 | 3 | Unfrozen state. | 1.053 |
| 19 | 1.2 | 5 | Unfrozen state. | 1.2630 |
| 19 | 1.2 | 7 | Unfrozen state. | 1.4310 |
| 19 | 1.2 | 9 | Unfrozen state. | 0.8416 |
| 19 | 1.3 | 0 | Unfrozen state. | 0.9772 |
| 19 | 1.3 | 1 | Unfrozen state. | 1.1660 |
| 19 | 1.3 | 3 | Unfrozen state. | 1.3270 |
| 19 | 1.3 | 5 | Unfrozen state. | 1.4740 |
| 19 | 1.3 | 7 | Unfrozen state. | 0.8912 |
| 19 | 1.3 | 9 | Unfrozen state. | 1.0530 |
| 19 | 1.4 | 0 | Unfrozen state. | 1.2410 |
| 19 | 1.4 | 1 | Unfrozen state. | 1.3990 |
| 19 | 1.4 | 3 | Unfrozen state. | 1.5439 |
| 19 | 1.4 | 5 | Unfrozen state. | 0.9214 |
| 19 | 1.4 | 7 | Unfrozen state. | 1.1320 |
| 19 | 1.4 | 9 | Unfrozen state. | 1.2750 |
| 19 | 1.5 | 0 | Unfrozen state. | 1.4630 |
| 19 | 1.5 | 1 | Unfrozen state. | 1.5740 |
| 19 | 1.5 | 3 | Unfrozen state. | 0.9600 |
| 19 | 1.5 | 5 | Unfrozen state. | 1.1760 |
| 19 | 1.5 | 7 | Unfrozen state. | 1.3330 |
| 19 | 1.5 | 9 | Unfrozen state. | 1.5540 |
| 19 | 1.6 | 0 | Unfrozen state. | 1.6288 |
| 19 | 1.6 | 1 | Unfrozen state. | 0.7681 |
| 19 | 1.6 | 3 | Unfrozen state. | 0.8238 |
| 19 | 1.6 | 5 | Unfrozen state. | 0.969 |
| 19 | 1.6 | 7 | Unfrozen state. | 1.1030 |
| 19 | 1.6 | 9 | Unfrozen state. | 1.2482 |
| 21 | 1.2 | 0 | Unfrozen state. | 0.8217 |
| 21 | 1.2 | 1 | Unfrozen state. | 0.8570 |
| 21 | 1.2 | 3 | Unfrozen state. | 1.0267 |
| 21 | 1.2 | 5 | Unfrozen state. | 1.1543 |
| 21 | 1.2 | 7 | Unfrozen state. | 1.3934 |
| 21 | 1.2 | 9 | Unfrozen state. | 0.8665 |
| 21 | 1.3 | 0 | Unfrozen state. | 0.9086 |
| 21 | 1.3 | 1 | Unfrozen state. | 1.0693 |
| 21 | 1.3 | 3 | Unfrozen state. | 1.2162 |
| 21 | 1.3 | 5 | Unfrozen state. | 1.418 |
| 21 | 1.3 | 7 | Unfrozen state. | 0.8895 |
| 21 | 1.3 | 9 | Unfrozen state. | 0.9986 |
| 21 | 1.4 | 0 | Unfrozen state. | 1.1402 |
| 21 | 1.4 | 1 | Unfrozen state. | 1.2690 |
| 21 | 1.4 | 3 | Unfrozen state. | 1.4783 |
| 21 | 1.4 | 5 | Unfrozen state. | 0.8915 |
| 21 | 1.4 | 7 | Unfrozen state. | 1.0660 |
| 21 | 1.4 | 9 | Unfrozen state. | 1.2053 |
| 21 | 1.5 | 0 | Unfrozen state. | 1.2890 |
| 21 | 1.5 | 1 | Unfrozen state. | 1.522 |
| 21 | 1.5 | 3 | Unfrozen state. | 0.7453 |
| 21 | 1.5 | 5 | Unfrozen state. | 0.7970 |
| 21 | 1.5 | 7 | Unfrozen state. | 0.948 |
| 21 | 1.5 | 9 | Unfrozen state. | 1.0603 |
| 21 | 1.6 | 0 | Unfrozen state. | 1.135 |
| 21 | 1.6 | 1 | Unfrozen state. | 0.7619 |
| 21 | 1.6 | 3 | Unfrozen state. | 0.8167 |
| 21 | 1.6 | 5 | Unfrozen state. | 0.9790 |
| 21 | 1.6 | 7 | Unfrozen state. | 1.0875 |
| 21 | 1.6 | 9 | Unfrozen state. | 1.237 |
| 23 | 1.2 | 0 | Unfrozen state. | 0.7862 |
| 23 | 1.2 | 1 | Unfrozen state. | 0.8621 |
| 23 | 1.2 | 3 | Unfrozen state. | 0.9906 |
| 23 | 1.2 | 5 | Unfrozen state. | 1.1340 |
| 23 | 1.2 | 7 | Unfrozen state. | 1.242 |
| 23 | 1.2 | 9 | Unfrozen state. | 0.8319 |
| 23 | 1.3 | 0 | Unfrozen state. | 0.9281 |
| 23 | 1.3 | 1 | Unfrozen state. | 1.0320 |
| 23 | 1.3 | 3 | Unfrozen state. | 1.1870 |
| 23 | 1.3 | 5 | Unfrozen state. | 1.356 |
| 23 | 1.3 | 7 | Unfrozen state. | 0.8518 |
| 23 | 1.3 | 9 | Unfrozen state. | 0.9608 |
| 23 | 1.4 | 0 | Unfrozen state. | 1.1190 |
| 23 | 1.4 | 1 | Unfrozen state. | 1.2310 |
| 23 | 1.4 | 3 | Unfrozen state. | 1.3780 |
| 23 | 1.4 | 5 | Unfrozen state. | 0.7256 |
| 23 | 1.4 | 7 | Unfrozen state. | 0.7865 |
| 23 | 1.4 | 9 | Unfrozen state. | 0.932 |
| 23 | 1.5 | 0 | Unfrozen state. | 0.9882 |
| 23 | 1.5 | 1 | Unfrozen state. | 1.048 |
| 23 | 1.5 | 3 | Unfrozen state. | 0.7293 |
| 23 | 1.5 | 5 | Unfrozen state. | 0.7968 |
| 23 | 1.5 | 7 | Unfrozen state. | 0.9555 |
| 23 | 1.5 | 9 | Unfrozen state. | 1.0230 |
| 23 | 1.6 | 0 | Unfrozen state. | 1.071 |
| 23 | 1.6 | 1 | Unfrozen state. | 0.7709 |
| 23 | 1.6 | 3 | Unfrozen state. | 0.8533 |
| 23 | 1.6 | 5 | Unfrozen state. | 0.9747 |
| 23 | 1.6 | 7 | Unfrozen state. | 1.0700 |
| 23 | 1.6 | 9 | Unfrozen state. | 1.141 |
| 25 | 1.2 | 0 | Unfrozen state. | 0.8083 |
| 25 | 1.2 | 1 | Unfrozen state. | 0.9069 |
| 25 | 1.2 | 3 | Unfrozen state. | 0.9813 |
| 25 | 1.2 | 5 | Unfrozen state. | 1.0840 |
| 25 | 1.2 | 7 | Unfrozen state. | 1.236 |
| 25 | 1.2 | 9 | Unfrozen state. | 0.8077 |
| 25 | 1.3 | 0 | Unfrozen state. | 0.9437 |
| 25 | 1.3 | 1 | Unfrozen state. | 0.9916 |
| 25 | 1.3 | 3 | Unfrozen state. | 1.0900 |
| 25 | 1.3 | 5 | Unfrozen state. | 1.246 |
| 25 | 1.3 | 7 | Unfrozen state. | 0.7007 |
| 25 | 1.3 | 9 | Unfrozen state. | 0.7480 |
| 25 | 1.4 | 0 | Unfrozen state. | 0.901 |
| 25 | 1.4 | 1 | Unfrozen state. | 0.9569 |
| 25 | 1.4 | 3 | Unfrozen state. | 0.9771 |
| 25 | 1.4 | 5 | Unfrozen state. | 0.7275 |
| 25 | 1.4 | 7 | Unfrozen state. | 0.7668 |
| 25 | 1.4 | 9 | Unfrozen state. | 0.9265 |
| 25 | 1.5 | 0 | Unfrozen state. | 1.0190 |
| 25 | 1.5 | 1 | Unfrozen state. | 1.0553 |
| 25 | 1.5 | 3 | Unfrozen state. | 0.7361 |
| 25 | 1.5 | 5 | Unfrozen state. | 0.8357 |
| 25 | 1.5 | 7 | Unfrozen state. | 0.9569 |
| 25 | 1.5 | 9 | Unfrozen state. | 1.0540 |
| 25 | 1.6 | 0 | Unfrozen state. | 1.119 |
| 25 | 1.6 | 1 | Unfrozen state. | 0.7514 |
| 25 | 1.6 | 3 | Unfrozen state. | 0.8723 |
| 25 | 1.6 | 5 | Unfrozen state. | 0.9790 |
| 25 | 1.6 | 7 | Unfrozen state. | 1.0630 |
| 25 | 1.6 | 9 | Unfrozen state. | 1.148 |
| 27 | 1.2 | 0 | Unfrozen state. | 0.7949 |
| 27 | 1.2 | 1 | Unfrozen state. | 0.9240 |
| 27 | 1.2 | 3 | Unfrozen state. | 0.9828 |
| 27 | 1.2 | 5 | Unfrozen state. | 1.0730 |
| 27 | 1.2 | 7 | Unfrozen state. | 1.196 |
| 27 | 1.2 | 9 | Unfrozen state. | 0.6974 |
| 27 | 1.3 | 0 | Unfrozen state. | 0.7435 |
| 27 | 1.3 | 1 | Unfrozen state. | 0.863 |
| 27 | 1.3 | 3 | Unfrozen state. | 0.9448 |
| 27 | 1.3 | 5 | Unfrozen state. | 0.9790 |
| 27 | 1.3 | 7 | Unfrozen state. | 0.7134 |
| 27 | 1.3 | 9 | Unfrozen state. | 0.7608 |
| 27 | 1.4 | 0 | Unfrozen state. | 0.8997 |
| 27 | 1.4 | 1 | Unfrozen state. | 0.9773 |
| 27 | 1.4 | 3 | Unfrozen state. | 1.024 |
| 27 | 1.4 | 5 | Unfrozen state. | 0.7321 |
| 27 | 1.4 | 7 | Unfrozen state. | 0.8075 |
| 27 | 1.4 | 9 | Unfrozen state. | 0.9259 |
| 27 | 1.5 | 0 | Unfrozen state. | 0.9626 |
| 27 | 1.5 | 1 | Unfrozen state. | 1.058 |
| 27 | 1.5 | 3 | Unfrozen state. | 0.7473 |
| 27 | 1.5 | 5 | Unfrozen state. | 0.8208 |
| 27 | 1.5 | 7 | Unfrozen state. | 0.9530 |
| 27 | 1.5 | 9 | Unfrozen state. | 1.0621 |
| 27 | 1.6 | 0 | Unfrozen state. | 1.096 |
| 27 | 1.6 | 1 | Unfrozen state. | 0.7617 |
| 27 | 1.6 | 3 | Unfrozen state. | 0.9086 |
| 27 | 1.6 | 5 | Unfrozen state. | 0.9797 |
| 27 | 1.6 | 7 | Unfrozen state. | 1.0776 |
| 27 | 1.6 | 9 | Unfrozen state. | 1.189 |

**S2 Table Cited data.**

| Dry density /(g·cm−3) | Water content /(%) | Temperature /（℃） | Thermal conductivity /(W·m-1·K-1) |
| --- | --- | --- | --- |
| 1.03 | 24 | -0.5 | 0.62 |
| 1.03 | 24 | -1 | 0.7 |
| 1.03 | 24 | -1.5 | 0.9 |
| 1.08 | 29 | -0.5 | 0.789 |
| 1.08 | 29 | -1 | 0.85 |
| 1.08 | 29 | -1.5 | 1.01 |
| 1.05 | 25 | -0.5 | 0.7 |
| 1.05 | 25 | -1 | 0.83 |
| 1.05 | 25 | -1.5 | 0.98 |
| 1.10 | 34 | -0.5 | 0.96 |
| 1.10 | 34 | -1 | 1 |
| 1.10 | 34 | -1.5 | 1.15 |
| 1.18 | 23 | -0.5 | 0.9 |
| 1.18 | 23 | -1 | 0.96 |
| 1.18 | 23 | -1.5 | 1.13 |
| 1.20 | 20 | -0.5 | 1 |
| 1.20 | 20 | -1 | 1.06 |
| 1.20 | 20 | -1.5 | 1.18 |
| 1.21 | 34 | -0.5 | 1.18 |
| 1.21 | 34 | -1 | 1.22 |
| 1.21 | 34 | -1.5 | 1.30 |
| 1.17 | 30 | -0.5 | 1.08 |
| 1.17 | 30 | -1 | 1.15 |
| 1.17 | 30 | -1.5 | 1.26 |
| 1.28 | 20 | -0.5 | 1.02 |
| 1.28 | 20 | -1 | 1.08 |
| 1.28 | 20 | -1.5 | 1.18 |
| 1.26 | 26 | -0.5 | 1.06 |
| 1.26 | 26 | -1 | 1.11 |
| 1.26 | 26 | -1.5 | 1.25 |
| 1.30 | 28 | -0.5 | 1.15 |
| 1.30 | 28 | -1 | 1.28 |
| 1.30 | 28 | -1.5 | 1.32 |
| 1.34 | 34 | -0.5 | 1.55 |
| 1.34 | 34 | -1 | 1.60 |
| 1.34 | 34 | -1.5 | 1.72 |
| 1.39 | 23 | -0.5 | 1.12 |
| 1.39 | 23 | -1 | 1.28 |
| 1.39 | 23 | -1.5 | 1.35 |
| 1.40 | 29 | -0.5 | 1.42 |
| 1.40 | 29 | -1 | 1.50 |
| 1.40 | 29 | -1.5 | 1.67 |
| 1.42 | 26 | -0.5 | 1.10 |
| 1.42 | 26 | -1 | 1.20 |
| 1.42 | 26 | -1.5 | 1.38 |
| 1.43 | 32 | -0.5 | 1.60 |
| 1.43 | 32 | -1 | 1.68 |
| 1.43 | 32 | -1.5 | 1.87 |
